# New Methodology of Human Health Express Diagnostics Based on Pulse Wave Measurements and Occlusion Test

**DOI:** 10.3390/jpm13030443

**Published:** 2023-02-28

**Authors:** Roman Davydov, Anna Zaitceva, Vadim Davydov, Daria Isakova, Maria Mazing

**Affiliations:** 1 Institute of Physics and Mechanics, Peter the Great St. Petersburg Polytechnic University, 195251 St. Petersburg, Russia; 2 Institute for Analytical Instrumentation of the Russian Academy of Sciences, 190103 St. Petersburg, Russia; 3 Institute of Biomedical Systems and Biotechnology, Peter the Great, St. Petersburg Polytechnic University, 195251 St. Petersburg, Russia; 4 Institute of Electronics and Telecommunications, Peter the Great St. Petersburg Polytechnic University, 195251 St. Petersburg, Russia; 5 Department of Photonics and Communication Lines, The Bonch-Bruevich Saint Petersburg State University of Telecommunication, 193232 St. Petersburg, Russia

**Keywords:** express diagnostics, pulse wave, blood, oxygen, laser radiation, optical sensor, wavelength, microcirculation, saturation

## Abstract

Nowadays, with the increase in the rhythm of life, the relevance of using express diagnostics methods for human health state estimation has significantly increased. We present a new express diagnostics method based on non-invasive measurements (the pulse wave shape, heart rate, blood pressure, and oxygen saturation of blood vessels and tissues). A feature of these measurements is that they can be carried out both in the hospital and at home. The new compact and portable optical hardware–software complex has been developed to measure tissue oxygen saturation. This complex makes it possible to reduce the measurement time from 60 min to 7–8 min, which reduces the likelihood of artifacts in the measurement process and increases its reliability. A new technique has been developed to carry out these measurements. A new optical sensor based on a line of charge-coupled devices has been developed to register a pulse wave in the far peripheral zone. The developed new technique for processing the pulse waveform and data on the oxygen saturation of hemoglobin in the blood and tissues allows a person to obtain additional information about their state of health independently. It will help to make conclusions about taking the necessary measures. This additional information allows the attending physician to provide more effective control over the course of treatment of the patient at any time since the methods of express diagnostics proposed by us have no restrictions on the number of applications. The functional state of more than 300 patients was studied. The results of various measurements are presented.

## 1. Introduction

In recent years, for many reasons, the issues of express diagnostics of the human health functional state in various situations have received increased attention [1,2,3]. The greatest preference in implementing the diagnosis of the functional state of human health is given to non-contact methods, especially in cases of their individual use outside a medical institution [4,5]. At the same time, most of the classic non-contact methods of express diagnostics (for example, by blood pressure, temperature, and heart rate), which a person can implement independently at the right time, have almost completely exhausted themselves in obtaining new information.

Unlike the considered methods of express diagnostics, pulse oximetry has not exhausted its possibilities for obtaining additional information about the state of human health in various situations, the potential of which has not yet been fully explored and studied [6,7]. Therefore, many scientists and specialists show increased attention to it. It is very important to have information about the quality of oxygen delivery to biological tissues for controlling the dynamics of body recovery processes [8]. Oxygen plays an essential role in aerobic life, being the final electron acceptor in mitochondria, from which energy in the form of adenosine triphosphate (ATP) is supplied to the entire body. The oxygen-rich gas mixture from the environment enters the lungs, from where it enters the blood from the alveoli. Further, it is transported by erythrocytes in a chemical bond with hemoglobin to tissues, where oxygen is then reduced to water in the mitochondria [9]. Monitoring the quality of the delivery, consumption, and supply of tissues with oxygen has long been one of the fundamental methods for assessing the state of the human body in clinical practice. Unfortunately, the most effective BGEA (balance gas–electrolyte analysis) method [10,11] for analyzing blood gases in dynamics is not intended for independent use by patients. Therefore, for personal use for these purposes, transmission pulse oximetry is used [12,13], which allows you to register a pulse wave and measure the pulse and oxygen saturation of hemoglobin in the blood. Modeling and analysis of the pulse waveform [14,15], assessment of its propagation velocity [16,17], and measurement of cardiac output based on it [18,19] help physicians assess the functional state of the cardiovascular and other human systems.

Numerous studies have shown that only information currently obtained using the method of pulse oximetry is not enough to make an unambiguous decision on determining the state of human health, especially in clinical practice. Therefore, optical tissue oximetry (OTO) has become more widespread for express diagnostics [20,21]. The advantage of OTO technology is that it involves the measurement of tissue oxygenation parameters regardless of arterial pulsation unlike the pulse oximetry method. It makes it possible to assess the saturation of tissues with oxygen both at a low amplitude of pulse fluctuations and in the complete absence of a pulse wave. In addition, the pulse oximetry method allows obtaining information about the only saturation level in the blood. At the same time, the studies show that information on the state of the microcirculation system in biological tissues is often needed both in different parts of the body and at different depths [22,23].

The use of laser radiation at various wavelengths from 410 to 940 nm in the OTO technology allows one to conduct spectral studies in biological tissues across multiple body parts. In addition, in the OTO technology, it is possible to control the power of laser radiation sources since there are no significant restrictions on their size, and, consequently, heat removal to prevent the possibility of overheating. In reflected and transmission pulse oximetry (SpO_2_), only two wavelengths are used: λ_1_ = 662 ± 2 nm and λ_2_ = 907 ± 2 nm. The two types of SpO_2_ sensors have significant design size limitations, especially when operating in reflection. It limits the power of the laser radiation, and, consequently, the signal-to-noise ratio of the recorded signal. In a reflected SpO_2_ sensor, this significantly limits the penetration depth of the laser radiation.

The state of the micro circular tissue systems is closely related to the main parameters of hemodynamics (including abnormalities). It affects both the metabolic resources of the body, which provide its adaptive capabilities, and the general functional state of a person. Therefore, exchange processes between blood and tissues are supplied due to blood circulation in vessels with a diameter of 2 to 200 microns (micro vessels). In this regard, the microcirculatory bed state is associated with the physiological mechanisms of circulatory system regulation and with the metabolic needs of organs and tissues, determining their normal functioning. 

Therefore, this problem’s relevance lies in that the microcirculation system is the most crucial part of the vascular bed. It plays a significant role in maintaining the homeostasis of all biological systems of the human body and in the main energy and metabolic processes in tissues, including the processes of the transcapillary transport of biological fluids [24,25].

Observation of the state of microcirculation allows obtaining information about the subtle mechanisms of the regulation of vascular–tissue relations, deviations in the work of which can indicate the presence of various pathological processes, both at an early and a late stage. Thus, several data have been accumulated on the study of microcirculation in diabetes mellitus [26], hypertension, venous insufficiency, and several other diseases [27,28], despite the variety of developed and used methods for studying microcirculation processes (such as transcutaneous oximetry [29] and laser Doppler flowmetry [30,31]). They have several significant drawbacks: when conducting research, the patient must be in a stationary position (lying down), the equipment is expensive, and requires qualified maintenance. In addition to these shortcomings, Doppler flowmetry adds a lengthy calibration procedure before each measurement (about 10 min). This does not allow using these technologies widely in everyday clinical practice both for assessing the functional state of the microvasculature and for diagnosing tissue blood flow disorders.

In recent decades, the OTO method has proven itself as a method of non-invasive monitoring of tissue oxygenation and diagnosis of microcirculation disorders. However, the existing technical implementations currently used to detect microcirculation disorders are still imperfect, making it difficult to obtain reliable information about the oxygen status of tissues, and, subsequently, they do not allow assessing the human health state.

One of the possible options for solving the problems is the use of new optical systems developed by us for the simultaneous registration of pulse wave signals and tissue oximetry, as well as methods for processing them with the combination of diagnostic information into a common database and comparison with data obtained earlier by other methods. It will allow obtaining new information about the state of the body from two systems that use new developments and new techniques, both for measuring and processing the recorded signals to interpret the integral signal. It is possible to compare and combine the obtained data since the measurements are carried out simultaneously. Previously, this was very difficult because measuring data for occlusion studies takes more than 60 min. The obtained new information and real-time data analysis from the two systems will allow a medical specialist to make a good decision about the personalized tactics of treating a patient with a possible violation of tissue microcirculation.

## 2. Materials and Methods

**Figure 1:**
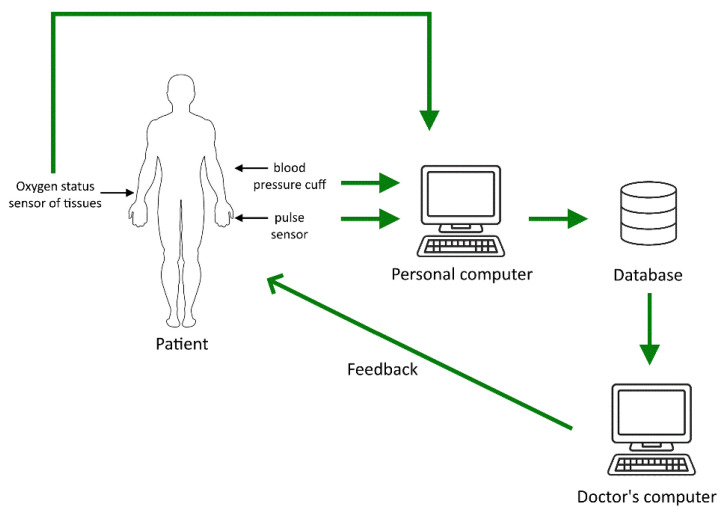
The scheme for monitoring the state of human health in express mode.

The main goal of all work in this area is to increase the reliability of data on the state of the human body, which are obtained after processing the results of measuring pulse waves and changes in tissue oxygen saturation. To successfully achieve the main target, we have developed a combined system for express diagnostics of the state of human health. Its block diagram is shown in Figure 1.

An optical sensor developed by us for registering a pulse wave, an optical system for studying tissue oximetry, and a standard device for measuring pressure are placed on the hands at three points for control. All measurements are carried out synchronously. Additionally, in the developed scheme, it is possible to use a sensor to control the CO_2_ content in the exhaled air from the lungs. These data can also be transferred to a personal computer. However, connecting this sensor will add additional difficulties that make it difficult for a person to independently cope with diagnosing the state of his body. Both hands are occupied by sensors for measurement (these hands must be motionless). It is undesirable to keep a sensor (sometimes heavy) for measuring CO_2_ on the weight of one of them. This may lead to failure in the measurement of other parameters. Furthermore, it is too hard for the person to perform all these measurements on his own. Therefore, we have not yet used it in our research, although we have it in our scientific laboratory.

All measured data are collected on a personal computer and processed using our developed methods and software. Further, several situations are possible. Figure 1 shows the most common of them. A personal computer is used to process information. Connecting a personal computer to a database on a remote server is necessary to ensure the complete analysis of the measured data on the state of human health. This database stores information about the results of studies of various patients. A doctor can use such information to analyze the measurements obtained using our devices. It will allow him to obtain additional information to identify the health problems.

Using the software developed by us, results are analyzed, considering the data in this database. After processing, information is transmitted to the doctor’s computer, which is also connected to a database. Additionally, a connection between the doctor and the patient is implemented, which allows the doctor to give real-time recommendations to the patient on taking measurements, for example, changing the measurement mode. In addition, the doctor can inform the patient in time about the danger associated with his state of health and recommend prompt action.

Sometimes, when taking measurements, there will be no real-time communication with the doctor. In such a situation, the patient can independently, based on the analysis of the measurement results and data from a personal computer (comparison of the data with the data in the database), conclude the significant change in his state of health and make an informed decision.

Such an approach to conducting express diagnostics of a person’s health status in personalized medicine is effective since a person can implement it independently in various situations without going to a medical institution without particular need.

### 2.1. Registration Methodology of a Pulse Wave and its Processing

A photodetector based on a charge-coupled device (CCD) is developed to register absorption signals from laser radiation at two wavelengths (λ_1_ = 662 nm and λ_2_ = 907 nm). The CCD line with horizontal charge transfer is used to increase the speed of the photodetector. In the new design of the CCD line, the photosensitive layer is thinned to 100 μm (standard value 300–350 μm) and doped with bromine (doping concentration 6%). Four hidden channels under the photosensitive layer are used to transmit information. This makes it possible to increase the signal-to-noise ratio several times during the registration of the pulse wave signal. The formation of a pulse wave signal is implemented in the form of steps corresponding to the levels of filling the photosensitive cells with a charge (quantization of the shape of the pulse wavefronts). This is a new form of registered pulse wave using laser radiation.

**Figure 2:**
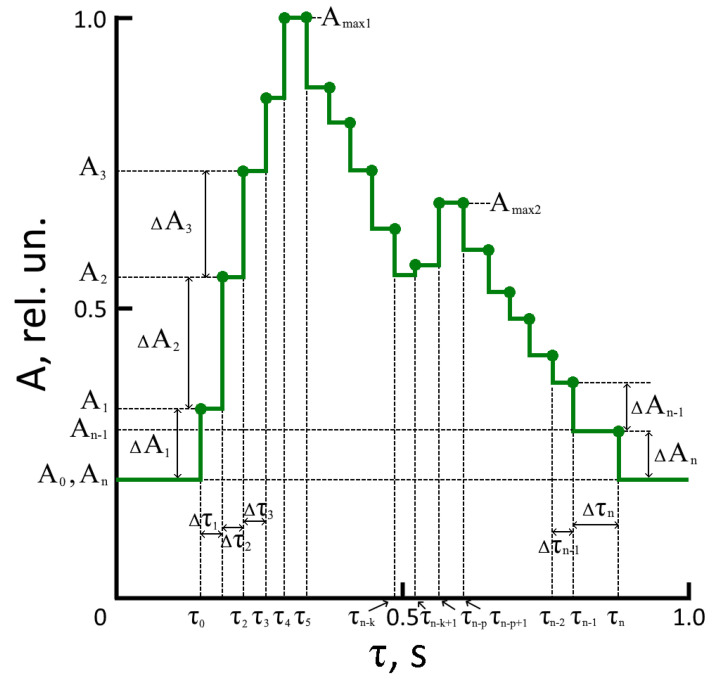
The pulse waveform (one period) recorded on the finger.

Figure 2 shows a sketch of one period of a pulse wave signal, recorded using a CCD line, where A_n_ is the amplitude n-th step of the pulse wave relative to the origin (zero level), Δτ_n_—its time duration. The time values in Figure 2 are presented for a good human heart rate — 60 beats per minute (pulse wave period is 1 s).

Unlike previously recorded laser radiation absorption signals using photodetectors or acoustic and inductive sensors, the process’s physics are displayed using a CCD. It is associated with the pulsation of the artery walls during the blood flow, the elasticity of arteries and veins, and the composition of the blood (hemoglobin concentration in the blood varies from person to person). The parameters of the steps (amplitude and duration) and their number in the pulse wave are different for different people. It is the scientific novelty of our work.

In devices for recording laser radiation absorption signals using photodetectors, acoustic or inductive sensors, steps in the form of a pulse wave are formed after processing the recorded signals by an analog-to-digital converter (ADC). The number of these steps in the pulse wave period depend on the clock frequency and the number of bits in the ADC. The greater the frequency and number of bits, the more steps in the form of a pulse wave. It helps to obtain more accurate values of the position on the time axis for maxima and minima. These data are necessary to solve several problems in diagnosing cardiac activity [14,15,16,17,18,19]. However, there is no information about the state of health in the parameters of these steps. In addition, any electronic device has a transfer function and its noise. The transfer function of the ADC introduces distortions into the pulse wave’s shape, and the ADC’s inherent noise worsens the signal-to-noise ratio and reduces the measurement accuracy. Using an ADC in the pulse wave signal processing circuit is not required when using a CCD line with horizontal charge transfer. This allows you to remove one of the factors of distortion of the pulse waveform and increase the signal-to-noise ratio. It is another novelty that distinguishes our work from the research of other scientists.

We have used the following technique to identify additional information in the pulse wave signal steps. The pulse wave signal must be divided into six intervals (two correspond to the rising front, two to the falling front, and two to the area of the maxima). It is a classical division of the pulse wave period, which is used regardless of the method of its registration [4,9,16,17,18,19,20,21]. One dependence is used to describe the rise fronts of a pulse wave (in amplitude from A_0_ to A_max1_ and from A_n-k_ to A_max2_), and another dependence is used for the decaying front (from A_max1_ to A_n-k_ and from A_max1_ to A_n_). In addition, we propose to use a separate function to study the neighborhoods of two maxima. A separate function for studying the maxima is necessary because the change in the nature of the pulse waveform and the parameters of the steps in the presence of several diseases in a person, for example, angina pectoris or arrhythmia, require a more detailed consideration of this part of the pulse wave (in the vicinity of two maxima) [32,33].

Since a new registration method of a pulse wave is used, the previously developed methods for processing pulse wave signals from the ADC output are unsuitable (there is no ADC circuit in our design). Therefore, we have developed new techniques for processing the pulse wave signal recorded in a new way. It is also a novelty of our work compared to previous ones. Previously, it was necessary to compensate for distortions introduced by the ADC into the pulse waveform. There is no ADC in the new design, and there are no such distortions. Steps in the form of a pulse wave signal correspond to a real change in the state of the human cardiovascular system. You can obtain additional information by processing the fronts of the pulse wave. Previously, there was no information on the fronts of the recorded pulse wave. The front parameters are determined by the parameters of the used ADC.

To assess the nature of the change in the pulse wave around the maxima, we propose to use the following function $P\left(t\right)$:

(1) $$P\left(t_{n}\right)=P\left(\sum_{n=m-p}^{n=m+p}\Delta \tau_{n}\right)=\left|\frac{A_{n}-A_{n-1}}{\Delta \tau_{n}}\right|,$$

where *m* is the step number of the corresponding maximum and *p* is the variable coefficient.

The following discrete function, $F_{1}\left(t\right)$, is used to describe the rising front of the pulse wave up to the first local maximum:

(2) $$F_{1}\left(t_{n}\right)=\sum_{n=1}^{n=m_{1}}\left(A_{n}\cdot \sum_{n=1}^{n=m_{1}}{\left[\frac{\Delta \tau_{n}}{\Delta \tau_{m_{1}}\frac{\left(m_{1}-1\right)}{p}}\right]}^{n}\right),$$

where *m*_1_ is the number of the step corresponding to the first local maximum.

In the area from the local minimum to the second local maximum, the following discrete function $F_{2}\left(t\right)$ is used:

(3) $$F_{2}\left(t_{n}\right)=\sum_{n=k_{2}}^{n=m_{2}}\left(A_{n}\cdot \sum_{n=k_{2}}^{n=m_{2}}{\left[\frac{\Delta \tau_{n}}{\Delta \tau_{m_{2}}\frac{\left(m_{2}-1\right)}{p}}\right]}^{n}\right),$$

where *m*_2_ is the number of the step corresponding to the second local maximum, and *k*_2_ is the number of the step corresponding to the second local minimum.

Function $Q\left(t\right)$ describes the segment of the pulse wave decline between the first local maximum and the local minimum, as well as after the second local maximum before the beginning of the rising front of the pulse wave to the first local maximum:

(4) $$Q\left(t_{n}\right)=A_{m}\cdot \left(exp\left(-\frac{\Delta \tau_{k}}{t}\cdot \frac{n-m}{m}\right)\frac{n-m}{p\left(A_{k-1}-A_{k}\right)}\right),$$

where the following restrictions are imposed on the values of t: τ_m_ < t, τ_k-1_ ≤ t < τ_k_. k is the number of the decline step.

In contrast to the classical methods of recording pulse waves using acoustic and induction sensors and photodetectors with analog outputs, the use of a CCD line allows several advantages. The registered signal is formed in digital form. In other cases of recording a pulse wave signal, it will need to be digitized using a transfer function, which introduces additional distortions both in the signal shape and in the positions of minima and maxima on the time axis.

Another feature of our method is the following. Each step at the pulse wave decay front, which is mainly determined by relaxation processes, is considered using (4) separately. Then, they are “crosslinked” at the boundaries of the steps, considering the duration of the time interval. This allows, when processing a pulse wave, the consideration of the characteristics of the body that are inherent in each person. This technique has not been previously implemented in other studies, considering relaxation processes in the analysis of the pulse wave decline segment is used for the first time.

### 2.2. Optical Tissue Oximetry

**Figure 3:**
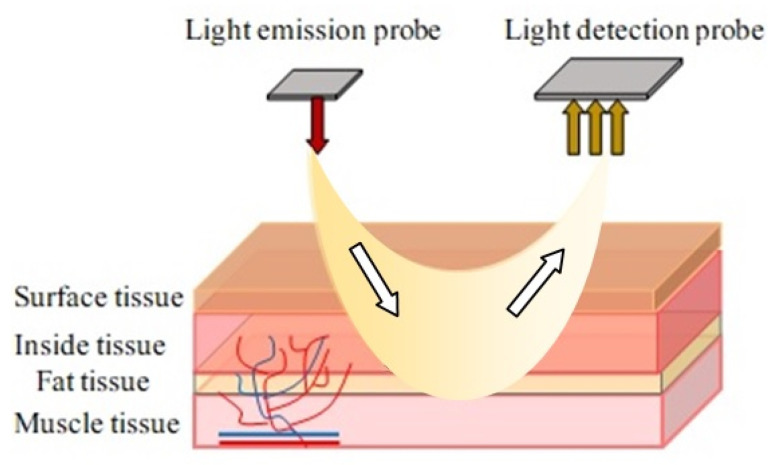
Structural diagram of a tissue oximeter sensor operating on a reflected signal.

Optical tissue oximetry is one of the most common diagnostic methods. The history of the active development of OTO technology dates to 1977, immediately after F. Jobsis published his important scientific work [34], which was devoted to studying the so-called “optical window” of biological tissue transparency. In various scientific papers, the values of the boundaries of this spectral range may differ slightly [35,36,37,38]. However, in general, it is considered that in the range between 600 nm and 950 nm, light passes through the tissue with the least difficulty, which provides the most significant penetration depth of the optical radiation in the biological tissue. In this case, the main absorbing chromophores in this optical range are the physiological fractions of the hemoglobin protein oxyhemoglobin and deoxyhemoglobin. The physical principle of the OTO technology lies in the difference in the spectral characteristics of various forms of hemoglobin. In contrast to the method of transmission pulse oximetry, in which the light transmitted through the tissues and blood is recorded (the LED and the detector are located on opposite sides), in all currently developed tissue oximeters, all optical components (emitter and photosensitive elements) are placed on the same side. In this mode of operation, the detector registers not the light that has passed but the light backscattered in biological tissues, which allows measurements to be made on different parts of the human body. In addition, the presence of several optical detectors in the design of an optical oximeter at different distances from the source makes it possible to assess tissue saturation at different depths, which is a great advantage of the technique for determining the presence of pathologies in biological tissues. A constructive representation of modern commercial tissue oximeters is presented in Figure 3.

Next, we will consider the principle of measurement briefly. Laser radiation through the human skin goes into tissues in all directions and is absorbed, in particular, by hemoglobin in the blood and myoglobin in the muscles. Optical sensors detect part of the backscattered light. The difference in light absorption at different wavelengths is used to determine the relative oxygenation level of the tissue being measured (since oxygenated and deoxygenated hemoglobin have different absorption spectra). Additionally, by taking measurements regardless of the presence of a pulse wave, modern tissue oximeters evaluate the oxygenation parameters of the general vascular bed. In contrast, the pulse oximetry method only allows obtaining information about arterial blood saturation.

Reflected pulse oximetry can be compared with OTO technology by the laser radiation registration principle. The SpO_2_ sensor for it can only be effectively used on a certain part of a person’s forehead and lower legs. This reduces its functionality compared to general relativity. Doctors could use the SpO_2_ sensor operating in the mode of reflection of laser radiation for additional brain monitoring in specialized medical institutions. In some cases, it is used in sports medicine when testing the work of the legs under load. So, it is more advisable to use a sensor with an absorption signal (transmission pulse oximetry) to study the work of the cardiovascular system with OTO technology.

**Figure 4:**
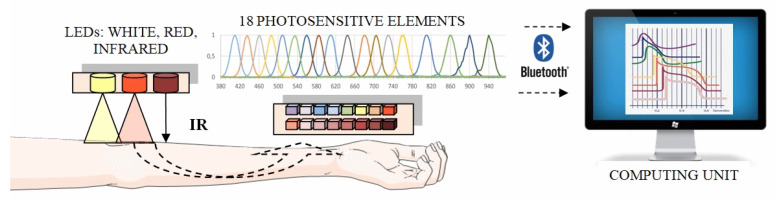
Structural diagram of a laboratory model of a hardware–software complex for analyzing the oxygen status of human tissues.

An optical hardware–software complex to implement our technique is developed for assessing the oxygen status of tissues, which is compact and portable. As the basic principle of its operation, a non-invasive method of optical tissue oximetry is chosen. In its reflection configuration, the radiation source and detector are located on the same side relative to the biological tissue under study while recording the light backscattered in the tissues. When developing the system, modern high-tech technical solutions and specialized information technologies, methods (algorithms, models) of data processing, and analysis (including machine learning methods) were used. Thus, the optical system consists of two main modules: electronic-sensory (measuring) and informational (computing) [39,40,41,42,43]. A personal computer runs specially designed programs for collecting, storing, and processing multidimensional data. The block diagram of the developed hardware and software complex is shown in Figure 4.

**Figure 5:**
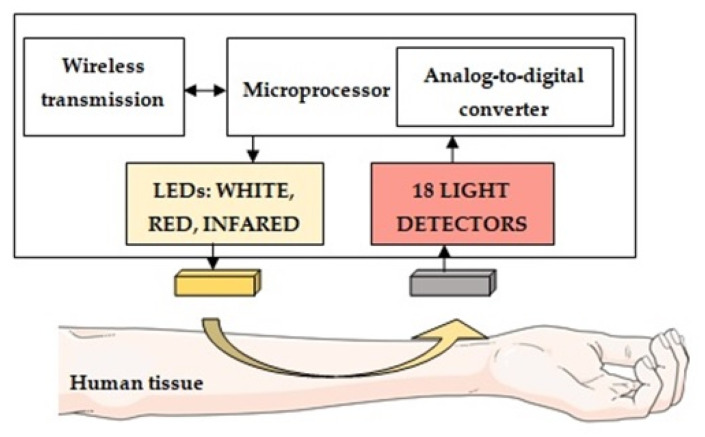
Multichannel spectrum analyzer architecture.

To obtain additional information about the oxygen state of tissues and the processes occurring in the studied microvasculature, we developed a multichannel system that operates in a wide range of optical radiation from 410 to 940 nm. Currently used research instruments operate the optical range from 600 to 960 nm [44,45,46,47]. The same wavelengths λ are needed to compare the results. The choice of wavelengths λ below 600 nm is due to the need to obtain additional information in the range of λ, where very little research has been carried out. For registration and processing of the reflected laser radiation, a multichannel integrated optical spectrum analyzer manufactured by AMS AG is used (Figure 5).

**Figure 6:**
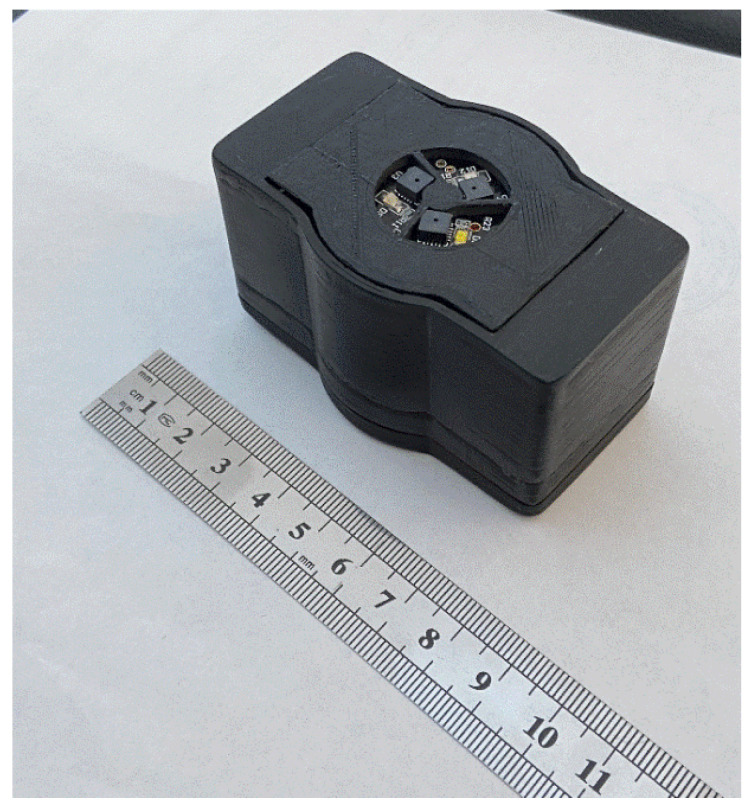
The appearance of the electronic sensor unit of the system, enclosed in a manufactured plastic case.

Eighteen channels with laser radiation of various wavelengths are used for research. Each channel has a spectral bandwidth of 20 nm. As radiation sources in the developed layout of the optical system, 3 SMD (Surface Mount Device)-type LEDs of different glow colors are used: cold white, red, and infrared (Kingbright, China). The SMD-type LEDs have several advantages over other semiconductor LEDs. These include the small size of the LED, low cost, and long service life. Although the white LED is not a source of a broadband emission spectrum (as, for example, incandescent lamps), the white SMD-type LEDs emit a fairly wide region of the visible range from blue to red light (range from 450 to 660 nm) with a pronounced dip in the blue-green color region (approximately 500 nm). The appearance of the measuring unit of the optical system is shown in Figure 6.

Bluetooth technology is chosen as a communication interface to connect the developed multichannel system with a computer. The wireless type of connection is necessary for the case of taking measurements when the patient is moving. The built-in battery allows the device to work continuously without recharging for up to 5 h.

The new design of the non-invasive electronic sensory system differs from the previously used ones in that it can be compactly placed on any part of the human body. The registered information is transmitted to the computing unit via the wireless data transmission module. Unlike previously developed systems, it is more efficient to record measurements at rest and when the patient performs functional and physical loads.

In the developed hardware–information complex, a new feature is the possibility of the simultaneous registration of radiation backscattered in tissues at once at eighteen wavelengths of the visible and near-infrared ranges of optical radiation. This makes it possible to reduce the measurement time to obtain the necessary information to 7–8 min compared to the 60 min or more required to obtain and interpret the same data on other equipment. Reducing the measurement time allows us to develop and apply a new technique for conducting simultaneous combined measurements. An analysis and comparison of the obtained results are provided for additional information about the state of human health. For a short time of 7–8 min, the probability of an artifact or external interference during measurement is much less than for a time of 60 min or more. It is much easier to maintain the desired state (without additional excitement) and posture for a person in the process of measuring (by 7–8 min) than after an hour or more. In addition, it is easier for a person to allocate a few minutes than an entire hour for the implementation of the measurement procedure. For these and other reasons, such measurements and comparisons have never been made before.

### 2.3. Experimental Study of the Human Health State 

The practical implementation of the techniques using new equipment was carried out on more than 300 patients. All patients consented to the study and were given information about their state of health in electronic form. This point is reflected in the informed consent they signed (forms are included in Supplementary Materials).

It should be noted that the new research equipment used is further intended for personal use by the patient (without the presence of a doctor) as part of developing a personalized medicine program. It is the advantage of our newly developed equipment and techniques compared to other instruments and techniques. Any patient can purchase this new equipment in the future. Examination with this equipment can be carried out without limiting the number of measurements. The measurements do not introduce any destructive factors into the state of blood vessels and tissues, as well as the work of the human cardiovascular system. Therefore, permission to conduct such measurements from state authorities in the Russian Federation is not required if people have signed consent.

Patients are divided into eight age groups (Table S1). Their health status, height, weight, and the presence of past diseases and pathologies known to them are considered. Table S1 also presents the results of interpreting the patient’s functional state of health obtained using methods for evaluating the two simultaneous measurement results. An assessment is made of the possibility of restoring blood flow in tissues to the initial values by studying microcirculation processes under an occlusive test. Three types of reactions to this functional load type are identified for 4 min after occlusion. The most typical reaction in all groups is restoring blood flow values to the original values. In addition, the following groups of patients are identified: with a significant decrease in blood flow values after functional exercise and a strong excessive recovery response.

To analyze pulse wave signals, we use several basic parameters, both classical and new ones, which we obtain after processing the fronts and maxima of the pulse wave signal recorded using new methods and equipment. The RI reflection index is defined as the ratio in percent of the diastolic component of the peripheral pulse wave’s height to the systolic component’s height (the index reflects the state of the tone of small arteries and the value of the pulse reflection wave). The stiffness index (SI) is calculated as the ratio of the patient’s height to the time interval Δt between the wave’s systolic and diastolic components (estimates the pulse wave’s speed).

The following parameters are new. The coefficient of pulse wave formation (K_z_) is determined by the ratio between the parameters of the diastolic and systolic maxima steps. It is related to the pulse wave formation process and the semilunar valves of the heart work. We propose to determine the value of K_z_ using the following relationship:

(5) Kz=1N ·(A4−A3Δτ4An−p−An−k+1τn−p−τn−k+1+A5−A4Δτ5An−p+1−An−pτn−p+1−τn−p+A6−A5Δτ6An−p+2−An−p+1τn−p+2−τn−p+1+A7−A6Δτ7An−p+3−An−p+2τn−p+3−τn−p+2),

where N is the number of steps formed in the diastolic maximum of the pulse wave (an even number for the considered pulse wave shape in Figure 2). The value of N can change for each pulse wave in the study of the state of human health. It is advisable to take the value of N as at most 12 when calculating this coefficient.

The rise front stability index (R_Fi_) of the pulse wave is determined by the change in dynamics (in time) in the ratio between the amplitudes of the histogram components and the variation in the period change δT_n_ between them:

(6) $$R_{Fi\left(n\right)}=\frac{\frac{F_{n}}{\Delta \tau_{n}}-\frac{F_{n+1}}{\Delta \tau_{n+1}}}{\delta T_{n}},$$

where n=1…K, and K is the number of columns in one period of the pulse wave at the rising front.

It is related to the dynamics of the work of the heart’s left ventricle and the rate of closing of the semilunar valves under various loads. It also characterizes the change in the elasticity of the aorta walls. The criterion of the state of health here is in the fulfillment of the following relation:

(7) $$R_{Fi\left(1\right)}=R_{Fi\left(2\right)}=R_{Fi\left(3\right)}=\ldots \;\equiv R_{Fi},$$

In case of non-fulfillment of relation (7), the value of R_Fi_ is calculated by the arithmetic mean of the values. Further, deviations of the current values R_Fi(n)_ from R_Fi_ at the steps of the rising pulse wavefront can be investigated, which depend on the elasticity of the aortic walls.

The two relaxation coefficients (systolic K_Rs_ and diastolic K_Rd_) of the pulse wave decline are determined by the changes in the slope angle on the decline or increase (if present) between two discrete states of the Q(t) function on the time axis as follows:

(8) $$K_{\mathrm{Rs}}=\frac{1}{m}\cdot \overset{m}{\underset{n=1}{\sum }}\arcsin\left(\frac{\left|Q_{n}-Q_{n+1}\right|t_{min}^{s}}{t_{n+1}-t_{n}}\right),$$

(9) $$K_{\mathrm{Rd}}=\frac{1}{m}\cdot \overset{m}{\underset{n=1}{\sum }}\arcsin\left(\frac{\left|Q_{n}-Q_{n+1}\right|t_{min}^{d}}{t_{n+1}-t_{n}}\right),$$

where *m* is the number of drops on the slopes of decline or increase in Q(t) function. The value of $t_{min}^{s}$ corresponds to minimum Q(t) on the decline slope, and $t_{min}^{d}$ on the increase slope.

They are related to the heart muscle work dynamics and features of the distribution of direct and are reflected from the semilunar valves pulse waves through the arteries and veins of a person, depending on their condition. This also characterizes the reaction resistance of the human circulatory system to a sharp impact on it and periodic loads. It has been experimentally established that the following ratio corresponds to the state of a person without deviations in the activity of the cardiovascular system:

(10) $$t_{min}^{s}=t_{min}^{d},$$

If (10) is not met, Formulas (8) and (9) use the values $t_{min}^{s}$ or $t_{min}^{d}$, which are closer to the interval for calculating the decline and increase in the function Q(t).

The measurements are made when each patient performs a functional load. It is an occlusive test, a short-term cessation of blood flow in the limb by pumping the tonometer cuff, followed by removing compression and observing the vessel’s tonic state restoration. The result of the occlusion test is the induction of post-occlusion hyperemia in the patient. It is a sharp increase in blood flow in the vessels after the opening of the blood flow, and the associated response of the blood vessels to the created conditions of hypoxia of the limb tissues. The intensity of response can characterize the person’s functional state.

The cuff occlusion test is carried out according to the generally accepted previously developed method as follows. When examining the upper limbs, the pulse oximeter readings are recorded in the region of the inner surface of the left hand’s phalanx’s index finger. The developed optical system is located on the forearm of the right hand. Recording of the indicators of all devices is carried out in parallel in the sitting position of each patient. Before the test, each patient has his blood pressure measured by an automatic tonometer. Afterward, a tonometer cuff is put on each patient’s shoulder (without pressurization), followed by turning on all measuring instruments. So, during the first 60 s, the preliminary initial level of peripheral blood flow, the basic level of pulse parameters, and saturation are recorded. After that, without interrupting the recording, the air is injected into the cuff to a value of 20–25 mmHg higher than systolic pressure, completely blocking even the internal arterial blood flow. Cuff occlusion lasts 3 min, after which the recording of readings continues for another 4 min (post-occlusion period). The measurements are taken continuously during the entire experiment with a frequency of every 10 s. The results obtained from all sensors are transmitted and stored on a computer to process the received data further.

**Figure 7:**
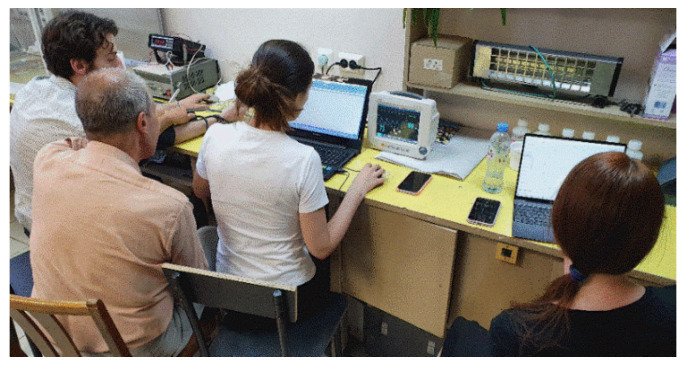
Testing patients.

Figure 7 shows the process of two measurements simultaneously in the laboratory. The patient (young male aged 25) is on the far left of the picture.

At the time of compression (occlusion) of the brachial artery, blood flow to the limb is suspended. With such exposure, there is a complete blockage of arterial blood flow in the vessels, in which artificially induced ischemia of the tissues of the upper limb occurs. At the end of arterial occlusion (cuff removal), blood flow in the limb is restored. Reactive post-occlusion hyperemia develops in the tissues, which manifests itself in a sharp increase in blood flow with a maximum filling of all micro vessels and small capillaries with blood, followed by restoration of the microcirculation system to its original level.

## 3. Results and Discussion

**Figure 8:**
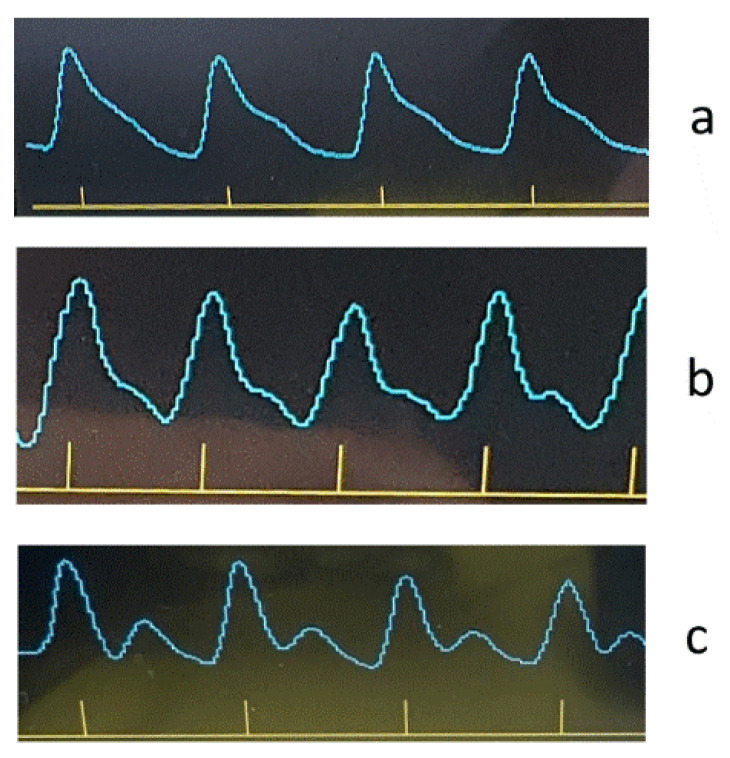
Registered pulse waves of the patients at the beginning of testing.

**Figure 9:**
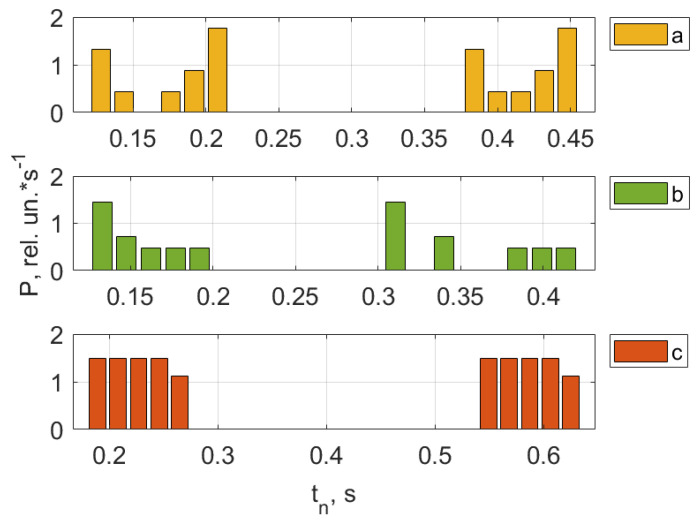
Pulse wave crest processing results. The first patient (**a**) is a 25-year-old man, (**b**) is a 28-year-old man, (**c**) is a 30-year-old man.

**Figure 10:**
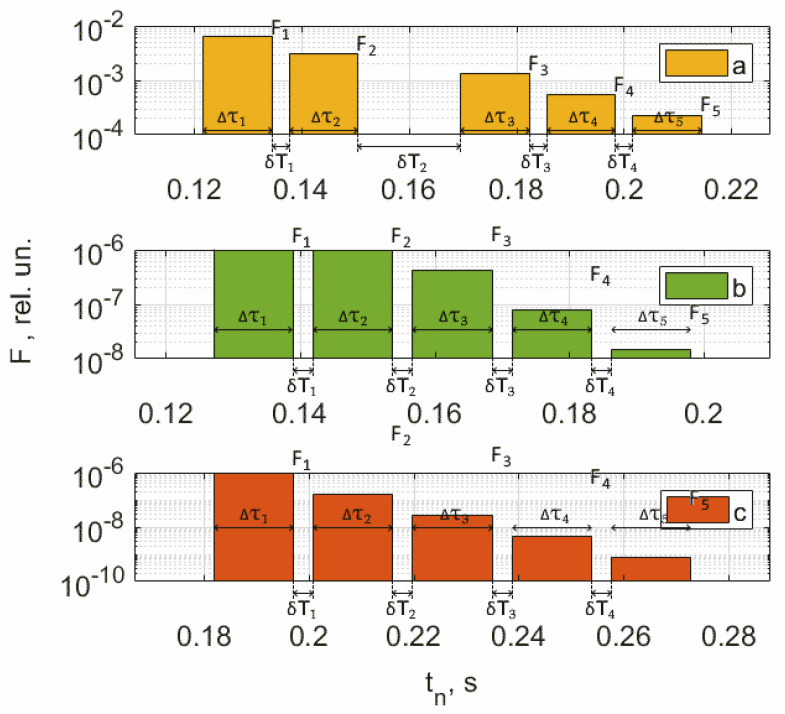
Results of processing the rise front of the pulse wave. The first patient (**a**) is a 25-year-old man, (**b**) is a 28-year-old man, (**c**) is a 30-year-old man.

**Figure 11:**
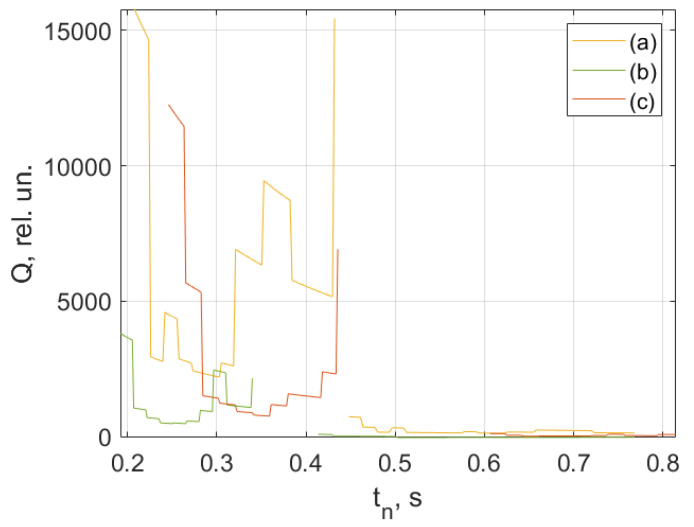
Results of processing the decline front of the pulse wave. The first patient (**a**) is a 25-year-old man, (**b**) is a 28-year-old man, (**c**) is a 30-year-old man.

As an example, Figure 8, Figure 9, Figure 10 and Figure 11 show the results of the analysis of a pulse wave by our proposed method. The first patient (Figure 8a) is a 25-year-old man, (Figure 8b) is a 28-year-old man, (Figure 8c) is a 30-year-old man.

Figure 9, Figure 10 and Figure 11 show the results of processing the first peak of the pulse wave using (1). The second peak (dicrotic wave) here was not processed since it was absent for this patient (a), indicating the semilunar valve’s poor functioning. It should be noted that this fiducial point is strongly influenced by vasomotor activity (such as vasodilation) and the compliance rate of upper arterial vessels. For another patient, (b), it is weakly expressed, leading to noticeable errors during processing since it is hard to accurately determine the maximum position on the time scale.

**Figure 12:**
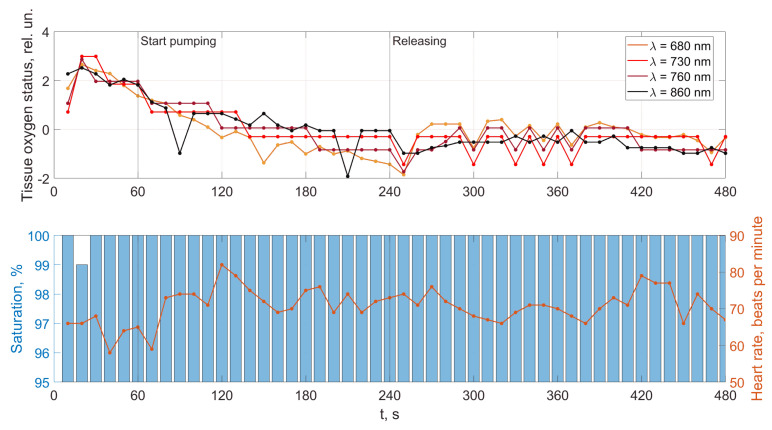
Dynamics of tissue oximetry parameters during the occlusion test for patient (a).

**Figure 13:**
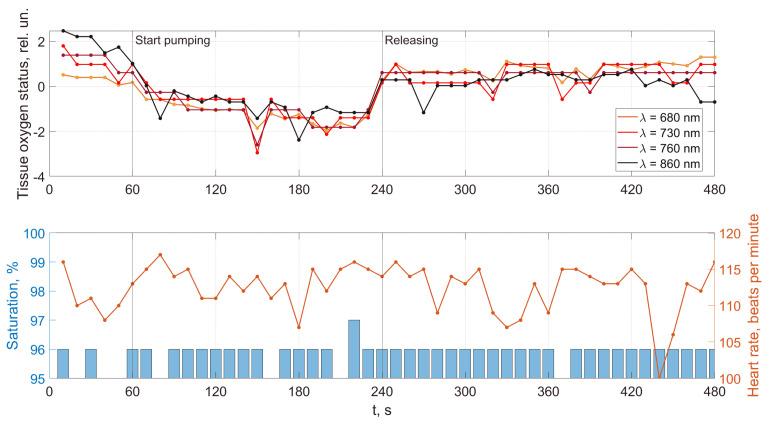
Dynamics of tissue oximetry parameters during the occlusion test for patient (b).

**Figure 14:**
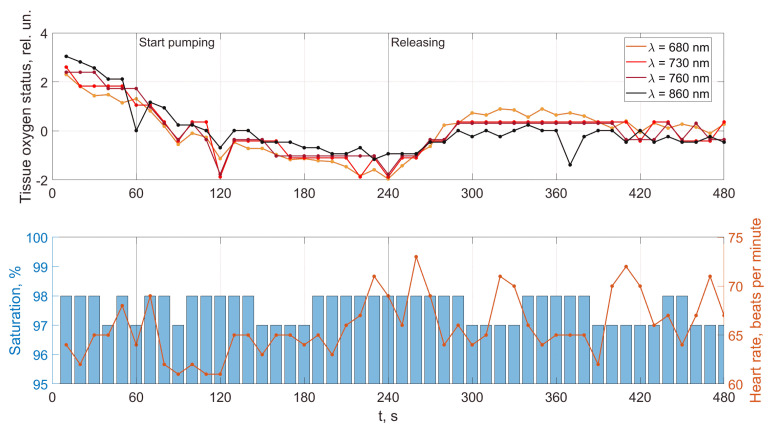
Dynamics of tissue oximetry parameters during the occlusion test for patient (c).

An analysis of the results obtained using a pulse wave shows that patient (a) has an unsatisfactory state of the cardiovascular system at the time of measurements, and measures must be taken. According to the research data, the cardiovascular system states of patients (b) and (c) are better. However, it is difficult to draw a definite conclusion. Additional information, in this case, is important. It is contained in data from 18 optical channels developed by the electron-optical system. The measurements were carried out at rest, during a stress test, and at the stage of restoration of blood flow. Additionally, the dynamics of changes in heart rate and SpO_2_ were analyzed. The obtained dependencies for the same three patients are shown in Figure 12, Figure 13 and Figure 14.

A comparison of the results in Figure 12, Figure 13 and Figure 14 shows that the response of the microvasculature to occlusal ischemia and occlusal stress is not always the same. There is a very different trend in the change in blood supply after occlusion, which indicates a different reaction of the organism of the patients to short-term ischemia and the created conditions of tissue hypoxia. At the same time, the severity of artificial ischemia of the limb, which can be observed by a gradual decrease in the values of the indicators of the sensors of the developed optical system during the occlusion action, is similar. The restoration of blood flow in the arteries of the limbs occurs with different dynamics, indicating that the microcirculatory bed’s reaction to the occlusion test is not the same. In this case, patient (b) has a more pronounced reactive post-occlusive hyperemia (Figure 14), manifested on the graphs as a sharp increase in the numerical values of the readings of the optical sensors immediately after the cuff is removed. On the contrary, for patient (a), blood filling in the limb almost does not increase after occlusion, which indicates the opposite type of response of the microvasculature to occlusive ischemia.

Furthermore, an important indicator is the blood flow reserve, which can be determined by the difference in sensor readings before and after occlusion. The blood flow reserve is a change in volumetric blood filling from minimal values during occlusion to maximal values during post-occlusive hyperemia. It can be determined by the difference in measurements of the optical sensors during and after the cuff test. So, for example, both a large and a reduced reserve of capillary blood flow can be detected. An increased value (above the norm) of this parameter may indicate a violation of blood circulation and a pathological narrowing of the vessels due to an inferior contraction of their walls. A reduced blood flow reserve indicator (below the norm) can be observed with vessel congestion.

In addition, the adaptive reserve of capillary blood flow characterizes the type of microcirculatory bed. So, this parameter can be used not only to assess the state of the blood microcirculation system, but also, as a criterion that allows reliably identifying individual typological features of human microcirculation and microhemodynamics. This is necessary for the subsequent assessment of the manifestation of the development of pathological processes.

The Supplementary Materials contain several tables. Table S1 presents information about patients who have provided informed consent to conduct the research. Tables S2 and S3 have statistically processed data from Table S1. This information is necessary to know the diversity of the studied population. The data are distributed in these tables by age groups with further division by weight and height.

A comparison and analysis of the results of measurements and patients’ data provide information for physicians: for example, examining what influence the incidence of COVID-19 had on the cardiovascular system state, or how these processes are related to human weight. When conducting long-term studies of people, it is possible to investigate the evolutionary processes of the influence of changes in the weight of a person with different heights on the work of the cardiovascular system. It would be helpful to conduct an impact analysis of past COVID-19 cases for different ratios in people between height and weight. In general, these data should be helpful for specialists. Table S4 presents the results of the conducted research on the health status of 306 people (including patient data presented in Figure 8, Figure 9, Figure 10, Figure 11, Figure 12, Figure 13 and Figure 14).

A combined analysis of the research results using pulse wave and the occlusion test allows us to make more informed conclusions about the functional state of patients. As an example, we consider three patients whose pulse waves and occlusion data are presented in Figure 8, Figure 9, Figure 10, Figure 11, Figure 12, Figure 13 and Figure 14 and Tables S1 and S4 (1–27, 2–47, 2–57).

For patient (a), at 100% saturation, there is a clear blockage of the semilunar valves. However, the patient’s blood pressure and pulse are within normal limits, and he feels well. This can be explained by the age of a person (25 years) when failures in the work of the cardiovascular system are still compensated. The occlusion data show a very poor restoration function of biological tissues after external impact, indicating progressive disease and confirming the problems identified using pulse wave data. This patient must be studied in a clinic to establish the causes of these deviations.

Patient (b), according to the study’s results, has problems with the work of the cardiovascular system. The semilunar valves close with failure, which in most cases is associated with a breakdown and a weakened immune system. The measurements were taken immediately after the patient defended his Ph.D. dissertation and were preceded by a large amount of psychological and emotional stress. When processing the form of the pulse wave, a single failure in the work of the heart is revealed. Occlusion studies confirm this failure. Studies of the oxygen saturation of tissues and the body’s response to exposure did not reveal the presence of deviations associated with the onset of diseases. The changes are more typical of overloading the body, which was previously noted when processing the pulse waveform. The patient needs rest. After that, it is necessary to examine them once again to establish the dynamics of changes.

Based on the comparison of the data obtained by the two methods, patient (c)’s functional state can be recognized as satisfactory. There are minor abnormalities in the saturation of tissues with oxygen. There can be many reasons for this. Conducting regular health monitoring using two methods can identify these causes and further take measures to eliminate them. It will allow us to fully implement the main target of our research, which is associated with an increase in the reliability of the results obtained on the state of human health compared with previously used methods.

## 4. Conclusions

The obtained results confirm the validity of new developments in non-invasive methods of express diagnostics, such as pulse oximetry and optical tissue oximetry. These developments made it possible to study the individual variability of hemodynamic parameters and evaluate microhemodynamic parameters during a short-term compression test (which previously was difficult to implement).

The developed technique provides the ability to implement measurements using new equipment for simultaneously recording a pulse wave and occlusion test so that the operation of one device has no significant effect on the operation of the other, and, consequently, on the measurement results. The obtained data on the functional state based on the measurement results of these two methods can be correctly compared (previously, data from measurements were not collected simultaneously, affecting comparison results). Such a combination of two methods for their simultaneous use in the express diagnostics of the functional state of the cardiovascular system is implemented for the first time.

The results of experimental studies have shown the proposed method’s effectiveness. It can be recommended for express monitoring of the oxygen supply to blood and tissues, including identifying individual typological features of human blood microcirculation. In addition, this method can be used to detect various destructive changes in the blood supply, which may indicate both chronic vascular diseases and the critical pathological changes caused by them in the human body that require urgent medical intervention. Such rapid monitoring of microcirculation and the main parameters of hemodynamics will help control the treatment process. Moreover, using it in the future may increase the number of patients seeking timely qualified medical care, thus reducing the number of complications after the disease and the time patients spend in hospitals.

A comparison of our results of the studies using new equipment (Table S4) with previously used methods and devices showed an increase in the reliability of interpretation of the functional state of health of patients from two to six times, depending on the type of disease and its stage. Using new methods and devices developed by us, we examined the condition of more than 300 people (patient data and investigation results are shown in Tables S1 and S4). Next, we took three samples of patients (100 people each) from different age groups. This is due to the fact that most scientists in the world use a scale of 100% to assess the different object’s state or situation, the concentration of substances, and other phenomena. We had new and previously used equipment at our disposal. All patients underwent the measurements discussed earlier in the article. Further, the analysis of the obtained results was carried out and conclusions were drawn about the cardiovascular system state and the general state of patient health. In the conclusions, possible diseases at an early stage or prerequisites for them (high probability of their onset) were noted. A group of people who do not have deviations in health was also established (according to the results of a study on the work of the cardiovascular system). All these people then underwent a comprehensive medical examination at the clinic of Peter the Great St. Petersburg University (St. Petersburg, Russia). When examining their state of health, methods and certified medical equipment were used (for example, an electrocardiogram; a 24-h monitor; in some cases, MRI, chemical and optical blood tests, and others). Doctors made the diagnosis in the polyclinic for health reasons. The data obtained from the results of the clinic survey were compared with those obtained using new devices and previously used ones, as well as information processing methods. The comparison was made on three sets of patients. Further, an assessment was made about increasing the results’ reliability. Since we consider different situations and classifications of the state of human health, we have a large interval for the spread of improvement in reliability from 2 to 6. The value of 2 mainly refers to the absence of deviations in the patient’s health. It is the hardest part. This is extremely important for people who have high workloads during their professional activities and require constant health monitoring. It can be difficult in some cases to distract them for a medical examination (for example, military, police, etc.).

Our studies have shown the availability of the developed methods and devices for personal use by a person, as well as the possibility of a more reliable analysis of the data obtained using previously obtained results (common database) and intelligent systems [39,40]. In addition, the devices we developed can work remotely, which is extremely important in various situations (moreover, in several situations, examining a patient online can be remotely controlled by a doctor and the measurement modes adjusted).

## Data Availability

Not applicable.

## Supplementary Materials

The following supporting information can be downloaded at: https://www.mdpi.com/article/10.3390/jpm13030443/s1, Table S1: The collected data from patients; Table S2: The statistics about COVID-19 illness for patients under study; Table S3: The relationship between height and weight for patients under study; Table S4: Research results. This part also contains patient consent forms, which they signed before conducting research in accordance with the approved ethical requirements of the Russian Federation.
