# Supplementary material for: New Methodology of Human Health Express Diagnostics Based on Pulse Wave Measurements and Occlusion Test
Source: J Pers Med. 2023 Feb 28;13(3):443. doi: 10.3390/jpm13030443 (PMC10052938; doi:10.3390/jpm13030443)
Supplement: Supplementary file 1 [file jpm-13-00443-s001.zip › jpm-2043277-supplementary.pdf]

Table S1. The collected data from patients.

Group and patient number	Age group	Age (full years)	Male	Female	weight, kg	height, cm	Past illnesses	
							Cardio-vascular	Covid-19 during 2022 year
1 - 1	18 - 25	18	+		78.5	177	No	Yes
1 - 2		18	+		82.5	183	No	Yes
1 - 3		18		+	65	174	No	No
1 - 4		18		+	67	175	No	Yes
1 - 5		19		+	71	177	No	No
1 - 6		19	+		79	183	No	Yes
1 - 7		19		+	76	180	No	Yes
1 - 8		19		+	57	166	No	No
1 - 9		19	+		67	174	No	Yes
1 - 10		19	+		69	172	No	Yes
1 - 11		19	+		89	193	No	Yes
1 - 12		19		+	68	181	No	No
1 - 13		20		+	75	180	No	No
1 - 14		20		+	73	179	No	Yes
1 - 15		20		+	68	176	No	Yes
1 - 16		20		+	62	177	No	Yes
1 - 17		20	+		79	185	No	Yes
1 - 18		21	+		80	179	Yes	No
1 - 19		21		+	52	164	No	No
1 - 20		22		+	51	162	No	Yes
1 - 21		22	+		59	166	No	Yes
1 - 22		23	+		64	177	No	Yes
1 - 23		23	+		98	201	No	Yes
1 - 24		24		+	78	186	Yes	No
1 - 25		25		+	81	185	No	Yes
1 - 26		25		+	47	153	No	Yes
**1 - 27**		**25**		**+**	**54**	**167**	**No**	**No**
1 - 28		25		+	58	172	No	Yes
1 - 29		25	+		76	178	No	Yes
1 - 30		25		+	67	177	No	Yes
1 - 31		25	+		78.5	184	No	No
2 - 32	26 - 39	26	+		84.5	180	Yes	Yes
2 – 33		26	+		76.5	181	No	No
2 – 34		26	+		77	180	No	No
2 – 35		26	+		76	176	No	Yes
2 – 36		27	+		85	190	Yes	Yes
2 – 37		27		+	67	173	No	Yes
2 – 38		27		+	65	170	No	Yes
2 – 39		27		+	66.5	172	No	No
2 – 40		27		+	77.5	174	Yes	No
2 – 41		27	+		76	182	No	Yes
2 – 42		27	+		89	185	Yes	Yes
2 – 43		27	+		75	180	No	Yes
2 – 44		27		+	76	165	Yes	No
2 – 45		27		+	57	173	No	No
2 – 46		28		+	47	156	No	Yes
**2 – 47**		**28**	**+**		**72**	**177**	**No**	**No**
2 – 48		28	+		87	193	No	Yes

2 – 49		29		+	58.5	156	Yes	Yes
2 – 50		29		+	62	171	No	Yes
2 – 51		29		+	63.5	172	No	Yes
2 – 52		29	+		89	178	Yes	Yes
2 – 53		29	+		82.5	188	No	No
2 – 54		30		+	65.5	182	No	Yes
2 – 55		30		+	56.7	175	No	No
2 – 56		30	+		63	174	No	No
**2 – 57**		**30**	**+**		**93**	**186**	**No**	**Yes**
2 – 58		30		+	48	163	No	Yes
2 – 59		30		+	52.5	164	No	Yes
2 – 60		31		+	54.4	165	No	Yes
2 – 61		31	+		96	181	Yes	No
2 – 62		31	+		79	181	No	No
2 – 63		31	+		76	180	No	Yes
2 – 64		31		+	67.5	178	No	Yes
2 – 65		32	+		78	182	No	No
2 – 66		32		+	56.5	168	No	No
2 – 67		32	+		67	173	No	Yes
2 – 68		32		+	62.5	172	No	Yes
2 – 69		33	+		87	178	Yes	Yes
2 – 70		33	+		76	177	No	Yes
2 – 71		33		+	68.5	164	Yes	No
2 – 72		33		+	65	173	No	No
2 – 73		33		+	63.5	172	No	Yes
2 – 74		34	+		78	161	Yes	No
2 – 75		34		+	66.4	171	No	Yes
2 – 76		34		+	63.5	172	No	Yes
2 - 77		34		+	71.5	174	No	No
2 – 78		34	+		87	169	Yes	No
2 – 79		35		+	63.5	175	No	Yes
2 – 80		35		+	64.5	174	No	Yes
2 – 81		35		+	78.5	171	Yes	Yes
2 – 82		36	+		76	172	No	No
2 – 83		36	+		83	179	Yes	Yes
2 – 84		37	+		87	187	No	No
2 – 85		37		+	72.5	176	No	Yes
2 – 86		37	+		77	180	No	No
2 – 87		37	+		78	182	No	Yes
2 – 88		38		+	76.5	171	No	Yes
2 – 89		38		+	72.5	168	No	No
2 – 90		38		+	73.5	170	No	Yes
2 – 91		39		+	72.5	173	No	Yes
2 - 92		39	+		87	175	Yes	Yes
3 - 93	40 - 49	40	+		67	174	No	No
3 – 94		40		+	68.5	163	Yes	Yes
3 – 95		40		+	65	173	No	No
3 – 96		40		+	75.5	164	Yes	Yes
3 – 97		40	+		67	175	No	No
3 – 98		40	+		87	185	No	No
3 – 99		40	+		79	176	No	Yes
3 - 100		40	+		92	178	Yes	Yes
3 – 101		41		+	74	167	No	Yes
3 – 102		41		+	72.5	166	No	No

3 – 103		41		+	84.5	170	Yes	No
3 – 104		41		+	76.5	173	No	Yes
3 – 105		41	+		78	179	No	Yes
3 – 106		42		+	67.5	175	Yes	No
3 – 107		42		+	66.4	174	Yes	Yes
3 – 108		42		+	59	172	No	No
3 – 109		42		+	54	168	No	No
3 – 110		42		+	67.5	163	Yes	Yes
3 – 111		43		+	67.5	176	No	Yes
3 – 112		43	+		79	173	Yes	Yes
3 – 113		43	+		82	175	Yes	Yes
3 – 114		43	+		79	180	No	No
3 – 115		43		+	62.5	174	No	No
3 – 116		43		+	75.5	167	Yes	Yes
3 – 117		43	+		87	185	No	No
3 – 118		43		+	86	179	No	Yes
3 – 119		43		+	95	178	Yes	Yes
3 – 120		43	+		88	182	No	Yes
3 – 121		43		+	67.5	177	No	No
3 - 122		44	+		79	179	Yes	Yes
3 – 123		44		+	69	166	Yes	Yes
3 – 124		44		+	57.5	172	No	No
3 - 125		44		+	86	170	Yes	Yes
3 – 126		45	+		79	179	No	No
3 – 127		45	+		87	176	Yes	Yes
3 – 128		45	+		82	178	No	Yes
3 – 129		45		+	67.5	153	Yes	No
3 – 130		45		+	57.7	157	No	Yes
3 – 131		46	+		75	177	No	Yes
3 – 132		46	+		87	176	Yes	No
3 – 133		46		+	88	177	Yes	Yes
3 – 134		46		+	82	174	Yes	Yes
3 – 135		46		+	74	179	No	No
3 – 136		46	+		87	188	Yes	No
3 – 137		46		+	78	167	Yes	Yes
3 – 138		47	+		79	167	Yes	Yes
3 – 139		47		+	54.5	172	No	No
3 – 140		47		+	68.5	175	Yes	Yes
3 - 141		47	+		78	171	Yes	Yes
3 – 142		47	+		79	176	No	No
3 – 143		48	+		88	176	Yes	Yes
3 – 144		48		+	65.5	174	No	No
3 – 145		48		+	63.5	176	No	Yes
3 – 146		49		+	78.5	178	Yes	Yes
3 – 147		49		+	77.4	169	Yes	Yes
3 – 148		49	+		79	167	Yes	Yes
3 – 149		49		+	64.3	170	Yes	No
3 – 150		49	+		68	175	No	Yes
3 – 151		49		+	68.2	164	Yes	No
3 – 152		49	+		86	175	Yes	Yes
3 - 153		49		+	56.5	171	No	Yes
4 - 154	50 - 59	50	+		79	172	Yes	No
4 – 155		50		+	58.8	163	Yes	Yes
4 – 156		50		+	57.5	165	No	No
4 – 157		50		+	58.5	164	No	No
4 – 158		50	+		86	176	Yes	Yes

4 – 159	50		+	74.5	167	Yes	Yes
4 – 160	50	+		78.5	178	No	Yes
4 – 161	50		+	67.5	156	Yes	Yes
4 – 162	50		+	63.4	162	No	No
4 – 163	50	+		89	173	Yes	Yes
4 – 164	50	+		92	175	Yes	Yes
4 – 165	50		+	75.3	165	Yes	Yes
4 – 166	50		+	72.5	171	No	No
4 – 167	51		+	79.5	173	Yes	Yes
4 – 168	51	+		85	179	No	Yes
4 – 169	51	+		96	178	Yes	No
4 – 170	51		+	62.5	158	Yes	Yes
4 – 171	51		+	59.5	167	No	Yes
4 – 172	52		+	75.5	172	Yes	No
4 – 173	52	+		79	173	Yes	Yes
4 – 174	52		+	69.5	177	No	Yes
4 – 175	52		+	72.5	165	Yes	No
4 – 176	52	+		93	179	Yes	Yes
4 – 177	52	+		86	178	No	Yes
4 – 178	52	+		102	176	Yes	Yes
4 – 179	52		+	58.5	173	No	No
4 – 180	52	+		93	175	Yes	Yes
4 – 181	52		+	67.5	165	Yes	No
4 – 182	52	+		89	175	Yes	Yes
4 – 183	52		+	62.5	173	No	No
4 – 184	52		+	68.5	168	Yes	Yes
4 – 185	52	+		87	174	Yes	Yes
4 – 186	52	+		93	176	Yes	Yes
4 – 187	53		+	54.5	172	No	No
4 – 188	53		+	73.5	169	Yes	Yes
4 – 189	53		+	76.5	170	Yes	Yes
4 – 190	53	+		87	182	No	No
4 – 191	53		+	78.5	172	Yes	Yes
4 – 192	53	+		94	176	Yes	No
4 – 193	53	+		81	175	No	Yes
4 – 194	53		+	65.5	170	No	Yes
4 – 195	54		+	74.5	167	Yes	Yes
4 – 196	54	+		100	177	Yes	Yes
4 – 197	54	+		98	174	Yes	Yes
4 – 198	54	+		87	174	Yes	Yes
4 – 199	54		+	68.5	171	Yes	No
4 – 200	54		+	65.4	167	Yes	Yes
4 – 201	54	+		87	179	No	Yes
4 – 202	54		+	63.5	158	Yes	No
4 – 203	55	+		89	171	Yes	Yes
4 – 204	55		+	67.5	168	Yes	Yes
4 – 205	55	+		80	176	No	Yes
4 – 206	55	+		89	173	Yes	No
4 – 207	55		+	56.5	167	Yes	Yes
4 – 208	56	+		102	176	Yes	No
4 – 209	56		+	61.5	168	No	Yes
4 – 210	56		+	57.5	171	No	Yes
4 – 211	56		+	74.5	164	Yes	No
4 – 212	56	+		87	172	Yes	Yes
4 – 213	56	+		79	175	No	Yes
4 - 214	56		+	66.5	165	Yes	Yes

4 – 215		56	+		83	172	Yes	No
4 – 216		57		+	74.5	167	Yes	No
4 – 217		57		+	56.5	163	No	Yes
4 – 218		57	+		86	172	Yes	Yes
4 – 219		58	+		88	173	Yes	Yes
4 – 220		58		+	72.5	166	Yes	No
4 – 221		58		+	56.5	164	No	Yes
4 – 222		58		+	64.5	166	Yes	No
4 - 223		59		+	68.5	165	Yes	Yes
5 - 224	60 - 69	60		+	72.3	165	Yes	Yes
5 – 225		60		+	71	166	Yes	No
5 – 226		60	+		87	173	Yes	Yes
5 – 227		60		+	61.5	168	No	No
5 – 228		60	+		89	174	Yes	Yes
5 – 229		60		+	66.5	159	Yes	No
5 – 230		61		+	68.5	162	Yes	No
5 – 231		61	+		94	177	Yes	Yes
5 – 232		61		+	72.6	168	Yes	Yes
5 – 233		61	+		83	178	No	No
5 – 234		61		+	67.5	165	Yes	Yes
5 – 235		62	+		79	169	Yes	Yes
5 – 236		62	+		92	178	Yes	Yes
5 – 237		62	+		90	174	Yes	Yes
5 – 238		62	+		83	172	Yes	No
5 – 239		62		+	54.5	165	No	Yes
5 – 240		62		+	67.5	163	Yes	Yes
5 – 241		63		+	57.5	166	No	Yes
5 – 242		63	+		79	167	Yes	No
5 – 243		63	+		78	170	Yes	Yes
5 – 244		63	+		89	175	Yes	Yes
5 – 245		63		+	66.5	172	Yes	No
5 – 246		64		+	72.5	171	Yes	Yes
5 – 247		64	+		79	174	Yes	Yes
5 – 248		64		+	59.5	163	Yes	No
5 – 249		64	+		80	178	No	Yes
5 – 250		64		+	75.5	171	Yes	Yes
5 – 251		64	+		87	173	Yes	No
5 – 252		65		+	74.5	168	Yes	Yes
5 – 253		65		+	65.5	170	No	Yes
5 - 254		65		+	76.5	169	Yes	No
5 – 255		65		+	59.5	171	No	Yes
5 – 256		65	+		78	173	Yes	Yes
5 – 257		65	+		89	176	Yes	No
5 – 258		66	+		91	177	Yes	Yes
5 – 259		66		+	62.5	165	Yes	No
5 – 260		66		+	76.5	166	Yes	Yes
5 – 261		66	+		94	176	Yes	No
5 – 252		66		+	57.5	171	No	Yes
5 – 263		67	+		90	174	Yes	Yes
5 – 264		67		+	79.5	172	Yes	No
5 – 265		67	+		87	169	Yes	Yes
5 – 266		67	+		79	168	Yes	Yes
5 - 267		67		+	67.5	164	Yes	Yes
5 – 268		67	+		88	173	Yes	Yes
5 – 269		67		+	89.5	177	Yes	No
5 – 270		68		+	83.5	174	Yes	Yes

5 – 271		69	+		95	180	Yes	Yes
5 – 272		68	+		81	180	No	No
5 – 273		69		+	76.5	168	Yes	Yes
5 – 274		69	+		87	173	Yes	Yes
5 – 275		69		+	74.5	167	Yes	No
5 – 276		69	+		87	168	Yes	Yes
5 - 277		69	+		93	176	Yes	No
6 – 278	70 – 74	70	+		87	172	Yes	Yes
6 – 279		70		+	61.5	169	No	No
6 – 280		70	+		92	177	Yes	Yes
6 – 281		70	+		87	176	Yes	Yes
6 – 282		71		+	67.5	171	Yes	No
6 – 283		71		+	78.5	170	Yes	Yes
6 – 284		71	+		76	174	No	No
6 – 285		71	+		89	176	Yes	Yes
6 – 286		72	+		73	173	No	Yes
6 – 287		72		+	73.5	167	Yes	No
6 – 288		72	+		86	170	Yes	Yes
6 – 289		72	+		88	172	Yes	No
6 – 290		73		+	60.5	167	No	Yes
6 – 291		73	+		78	171	Yes	Yes
6 – 292		73	+		82	173	Yes	No
6 – 293		74		+	67.5	174	No	Yes
6 – 294		74	+		104	181	Yes	Yes
6 – 295		74		+	88.5	168	Yes	No
6 – 296		74	+		80	178	No	Yes
6 – 297		74	+		87	167	Yes	Yes
6 - 298		74		+	78.5	172	Yes	No
7 - 299	75 - 79	75		+	85.5	170	Yes	No
7 - 300		75		+	63.5	171	No	Yes
7 - 301		77	+		79	168	Yes	No
7 - 302		79	+		86	173	Yes	No
7 - 303		79	+		87	174	Yes	Yes
7 - 304		79	+		76	175	No	No
7 - 305		79		+	77.5	162	Yes	No
8 - 306	>80	81	+		79	176	Yes	Yes

Table S2. The statistics about COVID-19 illness for patients under study.

Age group	Number of patients in the group	Percentage from the total number of patients, %	Number of patients not ill with COVID-19	%	Had COVID-19 without obvious symptoms	%	Seriously ill with COVID-19	%
18 - 25	31	10.1	3	9.7	23	74.0	5	16.3
26 - 39	61	20.0	9	14.7	24	39.4	28	45.9
40 - 49	61	19.8	8	13.1	18	29.5	35	57.4
50 - 59	70	22.9	10	14.3	22	31.4	38	54.3
60 - 69	54	17.6	8	14.8	9	16.6	38	68.6
70 – 74	21	7.0	4	19.0	7	33.3	10	47.7
75 - 79	7	2.3	2	28.6	3	42.8	2	28.6
>80	1	0.3	1	100	0	0	0	0

Table S3. The relationship between height and weight for patients under study.

Age group	M/F	Average weight, kg	Standard deviation, kg	Average height, cm	Standard deviation, cm
18-25	M	76,88	10,387	180,92	9,02
	F	65	10,01	173,94	8,56
26-39	M	80,60	7,45	179,55	6,41
	F	64,88	8,14	170,41	5,59
40-49	M	80,72	6,68	176,88	4,91
	F	70,60	9,96	170,64	6,05
50-59	M	88,53	6,63	175,29	2,60
	F	66,70	7,13	167,03	4,44
60-69	M	86,22	5,51	173,89	3,65
	F	69,51	8,39	167,63	4,01
70-74	M	85,31	7,93	173,85	3,72
	F	72	9,58	169,75	2,49
75-79	M	82	5,35	172,50	3,11
	F	75,5	11,14	167,67	4,93
>80	M	79	-	176	-
	F	0	-	0	-

Table S4. Research results.

Group and patient number	Restorative capacity of biological tissues			RI, %	SI, kg/ms	K_F, rel. un.	R_{Fi}, rel. un.	δT_Σ, s	K_{Rs}, rel. un.	K_{Rd}, rel. un
	insufficient	standard	excessive							
1 – 1		+		43.3	0.36	0.82	0.76	0.005	0.76	0.14
1 - 2		+		44.2	0.39	0.83	0.77	0.006	0.78	0.10
1 – 3		+		39.9	0.35	0.78	0.72	0.009	0.67	0.15
1 – 4			+	40.2	0.36	0.75	0.73	0.010	0.68	0.17
1 – 5		+		45.9	0.39	0.84	0.76	0.007	0.78	0.10
1 – 6		+		44.4	0.41	0.82	0.77	0.006	0.77	0.09
1 – 7		+		45.2	0.39	0.83	0.78	0.005	0.79	0.07
1 – 8		+		44.1	0.35	0.77	0.73	0.009	0.74	0.11
1 – 9	+			38.3	0.31	0.71	0.68	0.012	0.65	0.14
1 - 10		+		39.4	0.32	0.73	0.71	0.009	0.73	0.12
1 - 11		+		38.4	0.45	0.76	0.61	0.012	0.62	0.09
1 - 12	+			39.4	0.34	0.69	0.62	0.008	0.66	0.07
1 - 13		+		41.2	0.37	0.72	0.71	0.009	0.72	0.09
1 - 14		+		40.4	0.38	0.73	0.69	0.011	0.71	0.09
1 - 15		+		40.1	0.37	0.72	0.71	0.009	0.73	0.11
1 - 16			+	42.4	0.42	0.76	0.65	0.008	0.67	0.12
1 - 17		+		41.3	0.41	0.73	0.71	0.009	0.71	0.08
1 - 18			+	32.4	0.34	0.63	0.56	0.015	0.45	0.14
1 - 19		+		41.6	0.41	0.77	0.72	0.009	0.73	0.09
1 - 20		+		40.4	0.38	0.72	0.71	0.010	0.71	0.10
1 - 21		+		41.2	0.35	0.73	0.69	0.010	0.70	0.11
1 - 22		+		42.0	0.38	0.72	0.70	0.009	0.73	0.11
1 - 23		+		41.1	0.47	0.73	0.69	0.011	0.76	0.14
1 - 24			+	33.4	0.34	0.64	0.62	0.016	0.43	0.15
1 - 25		+		40.6	0.39	0.72	0.69	0.009	0.71	0.09
1 - 26		+		40.3	0.40	0.72	0.69	0.011	0.72	0.10
1 - 27		+		39.4	0.38	0.73	0.71	0.008	0.73	0.08
1 - 28		+		41.0	0.39	0.75	0.69	0.010	0.74	0.09

**1 - 29**	**+**			**0**	**----**	**0.27**	**0.68**	**0.023**	**0.17**	**0.18**
1 - 30		+		40.7	0.41	0.71	0.70	0.008	0.74	0.09
1 - 31		+		41.2	0.43	0.75	0.72	0.010	0.76	0.08
2 - 32			+	33.7	0.35	0.62	0.60	0.015	0.48	0.14
2 – 33		+		41.5	0.43	0.75	0.72	0.008	0.77	0.08
2 – 34		+		40.5	0.41	0.71	0.70	0.009	0.72	0.09
2 – 35		+		41.2	0.40	0.73	0.71	0.010	0.73	0.10
2 – 36			+	34.2	0.37	0.63	0.58	0.015	0.51	0.16
2 – 37	+			39.9	0.38	0.71	0.69	0.010	0.72	0.09
2 – 38		+		39.4	0.41	0.72	0.70	0.010	0.73	0.08
2 – 39		+		40.2	0.42	0.75	0.71	0.009	0.73	0.09
2 – 40	+			35.6	0.38	0.68	0.65	0.013	0.64	0.13
2 – 41		+		40.5	0.42	0.73	0.70	0.012	0.74	0.09
2 – 42			+	35.9	0.38	0.62	0.59	0.014	0.56	0.17
2 – 43		+		41.9	0.42	0.78	0.73	0.009	0.76	0.09
2 – 44			+	34.2	0.41	0.64	0.62	0.014	0.62	0.15
2 – 45		+		43.2	0.45	0.78	0.73	0.007	0.79	0.07
2 – 46		+		42.7	0.40	0.72	0.70	0.009	0.73	0.10
**2 – 47**			**+**	**38.7**	**0.37**	**0.58**	**0.46**	**0.009**	**0.45**	**0.11**
2 – 48		+		42.5	0.42	0.74	0.69	0.011	0.73	0.12
2 – 49	+			37.8	0.32	0.64	0.57	0.013	0.61	0.15
2 – 50		+		40.3	0.39	0.71	0.69	0.011	0.72	0.10
2 – 51		+		40.1	0.40	0.72	0.69	0.009	0.71	0.11
2 – 52			+	32.4	0.33	0.61	0.54	0.017	0.56	0.21
2 – 53		+		41.4	0.42	0.74	0.72	0.007	0.79	0.07
2 – 54	+			42.5	0.43	0.76	0.73	0.007	0.78	0.07
2 – 55		+		40.1	0.39	0.69	0.68	0.009	0.72	0.09
2 – 56		+		40.3	0.40	0.70	0.69	0.008	0.71	0.09
**2 – 57**		**+**		**42.9**	**0.42**	**0.88**	**0.87**	**0.005**	**0.88**	**0.09**
2 – 58	+			39.4	0.40	0.69	0.66	0.011	0.69	0.12
2 – 59		+		40.0	0.39	0.71	0.70	0.009	0.71	0.10
2 – 60		+		39.8	0.39	0.70	0.68	0.010	0.70	0.11
2 – 61			+	29.9	0.26	0.54	0.43	0.016	0.57	0.16
2 – 62		+		41.0	0.40	0.72	0.70	0.009	0.71	0.09
2 – 63		+		40.4	0.42	0.74	0.72	0.008	0.74	0.09
2 – 64	+			37.8	0.34	0.64	0.62	0.012	0.63	0.11
2 – 65		+		43.2	0.44	0.71	0.70	0.009	0.71	0.09
2 – 66		+		40.4	0.40	0.72	0.70	0.011	0.72	0.10
2 – 67	+			39.2	0.37	0.67	0.65	0.013	0.64	0.12
2 – 68		+		39.1	0.38	0.69	0.68	0.011	0.69	0.11
2 – 69			+	34.1	0.28	0.56	0.47	0.015	0.58	0.15
2 – 70		+		39.8	0.39	0.70	0.68	0.011	0.71	0.10
2 – 71			+	31.1	0.32	0.57	0.52	0.014	0.57	0.14
2 – 72		+		39.9	0.37	0.69	0.69	0.013	0.70	0.11
2 – 73		+		39.6	0.38	0.70	0.70	0.012	0.70	0.11
2 – 74			+	30.4	0.25	0.54	0.52	0.014	0.49	0.14
2 – 75		+		39.9	0.39	0.68	0.67	0.011	0.69	0.10
2 – 76		+		39.4	0.38	0.69	0.66	0.012	0.68	0.11
2 - 77		+		37.5	0.37	0.67	0.63	0.012	0.66	0.11
2 – 78			+	0	----	0.29	0.51	0.027	0.27	0.19
2 – 79		+		40.3	0.38	0.70	0.68	0.011	0.72	0.08
2 – 80		+		40.1	0.38	0.69	0.67	0.011	0.70	0.09
2 – 81			+	34.5	0.34	0.61	0.60	0.013	0.63	0.12
2 – 82		+		38.6	0.37	0.68	0.64	0.012	0.64	0.11
2 – 83	+			35.2	0.35	0.62	0.56	0.014	0.61	0.13
2 – 84		+		40.0	0.41	0.71	0.70	0.008	0.72	0.10

2 – 85		+		38.7	0.58	0.68	0.67	0.012	0.69	0.11
2 – 86		+		43.2	0.43	0.87	0.83	0.004	0.88	0.08
2 – 87	+			44.1	0.42	0.88	0.85	0.005	0.89	0.08
2 – 88		+		38.7	0.38	0.67	0.65	0.012	0.64	0.09
2 – 89		+		37.8	0.34	0.66	0.65	0.011	0.65	0.10
2 – 90		+		38.2	0.35	0.65	0.64	0.013	0.66	0.11
2 – 91		+		39.9	0.40	0.69	0.68	0.011	0.68	0.11
2 - 92			+	29.2	0.28	0.52	0.43	0.015	0.52	0.17
3 - 93		+		38.5	0.38	0.67	0.65	0.012	0.65	0.12
3 – 94			+	29.9	0.27	0.51	0.43	0.016	0.54	0.16
3 – 95		+		39.1	0.39	0.69	0.68	0.011	0.69	0.11
3 – 96			+	22.9	0.26	0.52	0.44	0.017	0.52	0.17
3 – 97		+		38.8	0.38	0.65	0.64	0.013	0.65	0.12
3 – 98	+			37.6	0.37	0.66	0.62	0.012	0.67	0.12
3 – 99		+		37.7	0.37	0.65	0.63	0.012	0.62	0.12
3 - 100			+	26.7	0.26	0.51	0.43	0.017	0.53	0.17
3 – 101		+		38.8	0.38	0.63	0.62	0.012	0.64	0.13
3 – 102	+			34.4	0.34	0.64	0.61	0.013	0.61	0.14
3 – 103			+	25.6	0.32	0.54	0.52	0.014	0.53	0.15
3 – 104		+		34.2	0.35	0.65	0.84	0.011	0.67	0.12
3 – 105		+		35.4	0.37	0.69	0.66	0.008	0.69	0.10
3 – 106			+	33.4	0.35	0.59	0.57	0.013	0.58	0.14
3 – 107			+	33.6	0.36	0.61	0.60	0.013	0.62	0.13
3 – 108		+		37.4	0.38	0.63	0.61	0.012	0.63	0.12
3 – 109		+		38.2	0.35	0.64	0.60	0.012	0.62	0.12
3 – 110	+			29.9	0.29	0.54	0.53	0.014	0.56	0.14
3 – 111		+		36.7	0.34	0.65	0.62	0.011	0.63	0.11
3 – 112			+	29.0	0.31	0.54	0.55	0.013	0.61	0.14
3 – 113			+	30.2	0.34	0.56	0.52	0.014	0.59	0.13
3 – 114		+		39.6	0.40	0.70	0.66	0.010	0.71	0.10
3 – 115		+		38.4	0.38	0.69	0.68	0.011	0.69	0.11
3 – 116			+	25.4	0.23	0.43	0.42	0.016	0.51	0.17
3 – 117		+		38.5	0.35	0.67	0.64	0.011	0.67	0.12
3 – 118	+			35.2	0.32	0.63	0.61	0.013	0.62	0.14
3 – 119			+	0	-----	0.24	0.32	0.024	0.32	0.19
3 – 120		+		35.6	0.37	0.63	0.63	0.012	0.62	0.14
3 – 121		+		37.6	0.38	0.67	0.65	0.011	0.64	0.13
3 - 122			+	35.1	0.34	0.61	0.57	0.014	0.59	0.15
3 – 123			+	31.1	0.30	0.52	0.51	0.015	0.54	0.16
3 – 124		+		34.5	0.32	0.67	0.66	0.011	0.71	0.12
3 - 125			+	0	-----	0.25	0.34	0.027	0.36	0.19
3 – 126		+		36.5	0.37	0.67	0.65	0.012	0.70	0.13
3 – 127			+	28.9	0.26	0.49	0.48	0.017	0.52	0.17
3 – 128		+		34.2	0.34	0.65	0.62	0.015	0.65	0.15
3 – 129			+	29.9	0.31	0.52	0.44	0.016	0.53	0.17
3 – 130		+		33.9	0.32	0.62	0.61	0.014	0.63	0.13
3 – 131		+		34.6	0.35	0.66	0.63	0.013	0.62	0.12
3 – 132			+	28.9	0.31	0.42	0.39	0.016	0.42	0.17
3 – 133			+	26.7	0.29	0.45	0.37	0.017	0.47	0.18
3 – 134	+			29.2	0.32	0.51	0.50	0.015	0.59	0.16
3 – 135		+		32.6	0.33	0.61	0.60	0.014	0.62	0.15
3 – 136		+		33.5	0.34	0.59	0.58	0.014	0.62	0.14
3 – 137			+	28.5	0.32	0.54	0.52	0.015	0.65	0.13
3 – 138	+			24.5	0.33	0.51	0.49	0.016	0.56	0.15
3 – 139		+		37.3	0.37	0.67	0.65	0.011	0.69	0.11
3 – 140		+		32.4	0.32	0.61	0.55	0.014	0.61	0.13

3 - 141			+	29.3	0.29	0.53	0.47	0.014	0.52	0.15
3 – 142		+		33.4	0.33	0.63	0.62	0.011	0.69	0.12
3 – 143			+	27.8	0.26	0.51	0.47	0.017	0.49	0.17
3 – 144		+		35.2	0.34	0.71	0.69	0.011	0.72	0.10
3 – 145		+		32.1	0.32	0.65	0.60	0.013	0.64	0.13
3 – 146		+		33.4	0.33	0.65	0.62	0.012	0.65	0.14
3 – 147			+	30.4	0.30	0.59	0.55	0.014	0.62	0.15
3 – 148			+	26.7	0.28	0.53	0.51	0.015	0.54	0.16
3 – 149	+			30.0	0.31	0.58	0.52	0.013	0.56	0.14
3 – 150		+		31.5	0.32	0.62	0.59	0.014	0.61	0.13
3 – 151			+	30.1	0.29	0.59	0.53	0.015	0.52	0.14
3 – 152			+	27.6	0.27	0.52	0.51	0.014	0.49	0.15
3 - 153		+		31.2	0.31	0.67	0.63	0.015	0.65	0.12
4 - 154			+	28.7	0.30	0.52	0.51	0.015	0.54	0.14
4 – 155		+		30.9	0.32	0.56	0.55	0.014	0.61	0.12
4 – 156		+		32.7	0.35	0.64	0.62	0.012	0.63	0.11
4 – 157		+		32.3	0.38	0.67	0.65	0.011	0.59	0.12
4 – 158			+	26.3	0.27	0.43	0.41	0.015	0.47	0.15
4 – 159		+		30.2	0.30	0.53	0.51	0.014	0.58	0.13
4 – 160		+		31.4	0.32	0.56	0.55	0.015	0.62	0.14
4 – 161			+	28.5	0.27	0.51	0.46	0.016	0.51	0.17
4 – 162	+			30.5	0.31	0.57	0.55	0.012	0.59	0.12
4 – 163			+	24.6	0.29	0.43	0.41	0.019	0.45	0.21
4 – 164			+	23.6	0.27	0.41	0.40	0.020	0.44	0.22
4 – 165	+			27.9	0.30	0.45	0.43	0.014	0.52	0.16
4 – 166		+		34.7	0.38	0.67	0.66	0.012	0.69	0.11
4 – 167			+	29.9	0.32	0.54	0.51	0.013	0.61	0.13
4 – 168		+		30.6	0.33	0.56	0.54	0.012	0.59	0.13
4 – 169			+	0	-----	0.26	0.21	0.032	0.24	0.26
4 – 170		+		27.8	0.30	0.49	0.48	0.015	0.47	0.18
4 – 171		+		32.4	0.35	0.61	0.60	0.011	0.67	0.12
4 – 172	+			29.6	0.29	0.59	0.58	0.013	0.61	0.13
4 – 173		+		30.5	0.32	0.53	0.52	0.013	0.59	0.14
4 – 174		+		34.2	0.34	0.63	0.62	0.012	0.62	0.13
4 – 175			+	30.2	0.31	0.56	0.51	0.014	0.53	0.14
4 – 176			+	27.1	0.25	0.43	0.41	0.016	0.52	0.15
4 – 177		+		30.2	0.34	0.45	0.44	0.017	0.54	0.16
4 – 178			+	20.6	0.22	0.31	0.30	0.021	0.46	0.23
4 – 179		+		31.5	0.34	0.67	0.64	0.012	0.70	0.11
4 – 180			+	25.4	0.27	0.37	0.35	0.018	0.41	0.19
4 – 181		+		29.9	0.31	0.57	0.52	0.013	0.65	0.13
4 – 182			+	24.5	0.26	0.46	0.43	0.016	0.51	0.18
4 – 183		+		34.2	0.34	0.67	0.66	0.013	0.71	0.13
4 – 184	+			30.9	0.31	0.58	0.53	0.015	0.61	0.15
4 – 185			+	28.9	0.30	0.56	0.55	0.014	0.62	0.14
4 – 186			+	26.4	0.25	0.45	0.42	0.017	0.47	0.19
4 – 187		+		32.7	0.32	0.67	0.66	0.014	0.71	0.12
4 – 188			+	25.3	0.26	0.51	0.43	0.018	0.46	0.19
4 – 189	+			28.8	0.30	0.59	0.57	0.013	0.65	0.14
4 – 190		+		35.6	0.38	0.67	0.65	0.012	0.71	0.11
4 – 191		+		30.4	0.29	0.56	0.52	0.014	0.59	0.15
4 – 192			+	0	-----	0.23	0.32	0.032	0.34	0.32
4 – 193	+			29.9	0.31	0.54	0.51	0.013	0.59	0.14
4 – 194		+		34.6	0.34	0.61	0.60	0.011	0.65	0.12
4 – 195	+			30.2	0.31	0.56	0.55	0.013	0.57	0.14
4 – 196			+	0	-----	0.22	0.29	0.034	0.41	0.33

4 – 197			+	21.3	0.22	0.32	0.31	0.030	0.45	0.27
4 – 198	+			28.5	0.29	0.51	0.45	0.017	0.51	0.16
4 – 199		+		30.2	0.31	0.57	0.55	0.013	0.59	0.14
4 – 200		+		31.5	0.32	0.61	0.58	0.014	0.67	0.14
4 – 201		+		30.9	0.32	0.59	0.51	0.016	0.56	0.16
4 – 202			+	25.4	0.25	0.49	0.48	0.015	0.53	0.16
4 – 203			+	0	-----	0.24	0.34	0.029	0.42	0.26
4 – 204		+		30.2	0.30	0.66	0.65	0.015	0.64	0.16
4 – 205		+		29.7	0.28	0.56	0.55	0.018	0.59	0.16
4 – 206			+	26.7	0.26	0.49	0.43	0.019	0.52	0.17
4 – 207		+		29.7	0.29	0.52	0.51	0.012	0.59	0.13
4 – 208			+	0	-----	0.21	0.25	0.026	0.38	0.24
4 – 209		+		30.3	0.31	0.58	0.55	0.013	0.57	0.12
4 – 210		+		30.2	0.32	0.59	0.56	0.012	0.61	0.11
4 – 211			+	28.5	0.29	0.49	0.48	0.014	0.53	0.13
4 – 212			+	27.3	0.26	0.45	0.44	0.014	0.53	0.14
4 – 213		+		30.1	0.31	0.56	0.54	0.013	0.56	0.13
4 - 214	+			29.7	0.30	0.61	0.60	0.012	0.65	0.12
4 – 215			+	27.2	0.25	0.51	0.45	0.014	0.53	0.14
4 – 216			+	28.1	0.23	0.51	0.44	0.015	0.47	0.15
4 – 217		+		32.4	0.33	0.67	0.64	0.011	0.71	0.10
4 – 218			+	28.4	0.27	0.51	0.44	0.015	0.48	0.15
4 – 219			+	24.6	0.25	0.45	0.41	0.017	0.43	0.23
4 – 220		+		29.9	0.31	0.61	0.59	0.014	0.65	0.13
4 – 221		+		30.4	0.32	0.65	0.62	0.013	0.67	0.12
4 – 222		+		30.1	0.31	0.64	0.59	0.014	0.66	0.13
4 - 223	+			29.8	0.27	0.53	0.51	0.013	0.57	0.15
5 - 224		+		30.1	0.31	0.64	0.62	0.013	0.67	0.13
5 – 225		+		30.5	0.32	0.66	0.63	0.013	0.68	0.12
5 – 226			+	27.3	0.24	0.45	0.43	0.016	0.53	0.15
5 – 227		+		32.5	0.33	0.63	0.62	0.013	0.67	0.13
5 – 228	+			0	-----	0.25	0.21	0.034	0.33	0.32
5 – 229	+			28.6	0.29	0.51	0.47	0.015	0.52	0.17
5 – 230			+	29.4	0.28	0.53	0.52	0.016	0.54	0.16
5 – 231			+	24.6	0.24	0.45	0.41	0.023	0.46	0.21
5 – 232		+		29.8	0.29	0.55	0.54	0.014	0.56	0.14
5 – 233		+		30.1	0.31	0.61	0..5<br>9	0.013	0.58	0.13
5 – 234			+	28.9	0.28	0.53	0.49	0.015	0.56	0.16
5 – 235			+	28.6	0.28	0.54	0.51	0.014	0.59	0.15
5 – 236			+	24.3	0.26	0.52	0.44	0.017	0.52	0.17
5 – 237			+	24.1	0.25	0.51	0.42	0.018	0.51	0.17
5 – 238			+	26.7	0.27	0.54	0.52	0.016	0.54	0.17
5 – 239		+		30.5	0.31	0.62	0.61	0.015	0.64	0.13
5 – 240			+	28.7	0.27	0.52	0.47	0.017	0.53	0.15
5 – 241		+		31.3	0.33	0.65	0.64	0.013	0.66	0.13
5 – 242			+	27.5	0.28	0.53	0.51	0.016	0.56	0.14
5 – 243		+		28.5	0.29	0.57	0.51	0.012	0.61	0.13
5 – 244	+			27.4	0.26	0.55	0.53	0.014	0.57	0.14
5 – 245		+		30.9	0.32	0.63	0.61	0.012	0.65	0.13
5 – 246		+		31.3	0.34	0.66	0.64	0.011	0.70	0.12
5 – 247	+			30.1	0.29	0.59	0.58	0.013	0.63	0.13
5 – 248			+	27.9	0.27	0.54	0.53	0.014	0.63	0.14
5 – 249		+		32.1	0.32	0.69	0.66	0.013	0.71	0.13
5 – 250		+		30.3	0.29	0.56	0.54	0.014	0.65	0.14
5 – 251			+	28.9	0.27	0.51	0.47	0.013	0.52	0.16

5 – 252	+			28.3	0.29	0.47	0.46	0.016	0.46	0.18
5 – 253		+		30.1	0.33	0.67	0.65	0.011	0.75	0.11
5 - 254	+			27.4	0.27	0.51	0.47	0.016	0.47	0.17
5 – 255		+		32.4	0.35	0.72	0.65	0.012	0.74	0.13
5 – 256		+		30.8	0.29	0.67	0.58	0.015	0.66	0.14
5 – 257			+	25.7	0.27	0.51	0.45	0.018	0.54	0.19
5 – 258			+	25.2	0.26	0.52	0.44	0.019	0.52	0.20
5 – 259		+		29.9	0.28	0.61	0.59	0.013	0.56	0.14
5 – 260		+		31.6	0.32	0.67	0.66	0.012	0.67	0.13
5 – 261			+	0	-----	0.25	0.31	0.035	0.41	0.32
5 – 252		+		32.4	0.33	0.67	0.62	0.011	0.72	0.09
5 – 263			+	24.6	0.23	0.45	0.39	0.023	0.43	0.25
5 – 264			+	27.9	0.28	0.54	0.48	0.014	0.57	0.15
5 – 265			+	0	----	0.23	0.24	0.026	0.41	0.31
5 – 266			+	26.7	0.27	0.51	0.43	0.015	0.47	0.21
5 - 267		+		29.9	0.31	0.57	0.56	0.013	0.65	0.14
5 – 268	+			28.7	0.30	0.53	0.48	0.014	0.57	0.15
5 – 269		+		31.5	0.32	0.63	0.62	0.015	0.64	0.14
5 – 270			+	29.5	0.28	0.52	0.44	0.018	0.54	0.18
5 – 271			+	0	-----	0.25	0.22	0.028	0.39	0.35
5 – 272		+		34.2	0.35	0.72	0.71	0.012	0.78	0.13
5 – 273	+			30.2	0.31	0.62	0.59	0.013	0.65	0.15
5 – 274			+	27.3	0.26	0.52	0.46	0.018	0.54	0.17
5 – 275		+		30.1	0.29	0.59	0.54	0.015	0.62	0.14
5 – 276			+	26.5	0.26	0.51	0.44	0.018	0.54	0.19
5 - 277			+	26.1	0.25	0.52	0.48	0.019	0.52	0.18
6 – 278			+	24.5	0.26	0.54	0.46	0.017	0.56	0.18
6 – 279		+		31.3	0.34	0.68	0.66	0.013	0.74	0.15
6 – 280			+	0	-----	0.26	0.25	0.023	0.43	0.29
6 – 281	+			27.5	0.25	0.52	0.46	0.018	0.54	0.18
6 – 282		+		29.9	0.31	0.59	0.54	0.014	0.65	0.14
6 – 283		+		28.9	0.30	0.58	0.56	0.013	0.64	0.15
6 – 284		+		34.4	0.37	0.71	0.69	0.011	0.79	0.13
6 – 285			+	30.1	0.29	0.65	0.61	0.014	0.67	0.14
6 – 286		+		35.1	0.37	0.67	0.64	0.011	0.80	0.13
6 – 287	+			30.3	0.29	0.61	0.54	0.014	0.65	0.14
6 – 288			+	28.6	0.26	0.53	0.45	0.018	0.54	0.17
6 – 289			+	27.4	0.27	0.55	0.48	0.017	0.56	0.18
6 – 290		+		32.3	0.33	0.65	0.62	0.013	0.71	0.13
6 – 291	+			28.9	0.29	0.57	0.53	0.014	0.62	0.14
6 – 292		+		31.2	0.29	0.58	0.55	0.016	0.59	0.16
6 – 293			+	31.8	0.32	0.57	0.53	0.014	0.59	0.14
6 – 294	+			0	-----	0.21	0.19	0.032	0.31	0.35
6 – 295			+	0	-----	0.25	0.23	0.029	0.41	0.34
6 – 296		+		29.7	0.27	0.57	0.52	0.015	0.61	0.16
6 – 297			+	24.5	0.25	0.49	0.43	0.021	0.51	0.22
6 - 298		+		28.9	0.29	0.61	0.54	0.014	0.63	0.15
7 - 299	+			27.8	0.28	0.54	0.52	0.016	0.57	0.17
7 - 300		+		31.0	0.32	0.67	0.64	0.013	0.71	0.14
7 - 301	+			28.6	0.25	0.52	0.45	0.017	0.54	0.19
7 - 302	+			27.4	0.26	0.53	0.50	0.018	0.54	0.19
7 - 303	+			28.1	0.27	0.51	0.45	0.017	0.54	0.20
7 - 304		+		31.4	0.32	0.58	0.52	0.016	0.59	0.15
7 - 305	+			25.2	0.25	0.51	0.43	0.019	0.55	0.19
8 - 306		+		28.5	0.28	0.56	0.45	0.017	0.59	0.16
